# Bioactivity studies of porphyrinoids against microsporidia isolated from honeybees

**DOI:** 10.1038/s41598-020-68420-5

**Published:** 2020-07-14

**Authors:** Katarzyna Buczek, Mariusz Trytek, Kamil Deryło, Grzegorz Borsuk, Katarzyna Rybicka-Jasińska, Dorota Gryko, Małgorzata Cytryńska, Marek Tchórzewski

**Affiliations:** 1 Department of Industrial and Environmental Microbiology, Institute of Biological Sciences, Faculty of Biology and Biotechnology, Maria Curie-Skłodowska University, Akademicka 19, 20-033 Lublin, Poland; 2 Department of Molecular Biology, Institute of Biological Sciences, Faculty of Biology and Biotechnology, Maria Curie-Skłodowska University, Akademicka 19, 20-033 Lublin, Poland; 3 Institute of Biological Basis of Animal Production, Faculty of Biology, Animal Sciences and Bioeconomy, University of Life Sciences in Lublin, Akademicka 13, 20-950 Lublin, Poland; 4 Institute of Organic Chemistry, Polish Academy of Sciences, Kasprzaka 44/52, 01-224 Warsaw, Poland; 5 Department of Immunobiology, Institute of Biological Sciences, Faculty of Biology and Biotechnology, Maria Curie-Skłodowska University, Akademicka 19, 20-033 Lublin, Poland

**Keywords:** Chemical modification, Biological fluorescence, Permeation and transport, Cellular imaging, Mechanism of action, Antifungal agents, Antiparasitic agents, Pathogens, Fungal infection

## Abstract

Microsporidian infections are dangerous to honeybees due to the absence of an efficient treatment for nosemosis. In the present work, the abilities of several porphyrins to directly inactivate microsporidia derived from *Nosema*-infected honeybees were studied in vitro. Amide derivatives of protoporphyrin IX (PPIX) conjugated with one and two amino acid moieties were synthesized, and their activities were compared with those of two cationic porphyrins, TMePyP and TTMePP. The most active porphyrins, PP[Lys-Asp]_2_, PP[Lys-TFA]_2_, PP[Asp(ONa)_2_]_2_ and PP[Lys-Lys]_2_ at concentrations as low as 10–50 µM exerted significant effects on microsporidia, reducing the number of spores by 67–80% compared to the control. Live-cell imaging of the spores treated with porphyrins showed that only 1.6% and 3.0% of spores remained alive after 24 h-incubation with 50 µM PP[Asp(ONa)_2_]_2_ and PP[Lys-Asp]_2_, respectively. The length of the amino acid side chains and their identity in the PPIX molecules affected the bioactivity of the porphyrin. Importantly, the irradiation of the porphyrins did not enhance their potency in destroying *Nosema* spores. We showed that the porphyrins accumulated inside the living spores but not inside dead spores, thus the destruction of the microsporidia by non-metallated porphyrins is not dependent on photosensitization, but is associated with their active transport into the spore cell. When administered to honeybees in vivo, PPIX[Lys-TFA]_2_ and PPIX[Lys-Lys]_2_ reduced spore loads by 69–76% in infected individuals. They both had no toxic effect on honeybees, in contrast to zinc-coordinated porphyrin.

## Introduction

Microsporidia are a large group of eukaryotic obligatory intracellular parasites that form single-cell spores and can complete their life cycle only within an infected host cell. So far, ~ 1400 microsporidian species representing 200 genera have been reported^1^. Microsporidia are in the kingdom Fungi, and chitin is a major component of the inner layer of their spore wall. An electron-dense outer exospore comprises the second layer of the cell wall^2–4^. Microsporidia are characterized by their unique metabolism and the absence of certain elements common to eukaryotic cells, i.e., mitochondria, peroxisomes, and the classic Golgi apparatus^3–5^. The interior of the spore is filled with sporoplasm and a mass of vesicular tubules, which are structurally homologous to the Golgi apparatus. Microsporidia contain a unique invasion apparatus that consists of a polar tube that coils around the sporoplasm and ends at an anchoring disc in the apical part of the spore. A polar tube is required for cell invasion, which occurs via injection of the spore content into the host cell^6^. All of these features are associated with adaptation to the parasitic lifestyle^3^. Microsporidia are able to survive outside a host cell but can exist only as metabolically inactive spores^7^.

Microsporidia cause many contagious diseases commonly known as microsporidiosis, and they can infect a wide range of organisms from invertebrates to vertebrates, including humans^8,9^. Microsporidian infections caused by *Nosema apis* and *Nosema ceranae* induce a disease named nosemosis in honeybees. *Nosema apis* is a long-known parasite of *Apis mellifera*, whereas *N. ceranae* is presumed to have more recently undergone a host shift from the Asian honeybee *Apis cerana* to the European honeybee *A. mellifera*^10–12^. Since 2005, surveys have shown that *N. ceranae* is the dominant microsporidium infecting honeybees in many parts of the world^10,13,14^. This parasite has been emerging worldwide and presents a high prevalence in honeybee colonies, contributing to economic losses. Therefore, developing compounds that can efficiently control microsporidia while simultaneously being harmless to humans and the environment is of great importance.

To date, there are no compounds that can control nosemosis and be safely used in managing honeybee colonies^15^. Fumagillin, the only compound that is effective in the treatment of the disease^16^, is currently banned in Europe because of its fairly high toxicity to humans^15,17^. In addition, since benzimidazoles and nitroimidazoles have activity against microsporidia in invertebrates^18^, synthetic antibiotics such as albendazoles, ornidazoles, tinidazoles, and metronidazoles were also tested against nosemosis^19^. Two of them (metronidazol and tinidazole) were effective in inhibiting the proliferation of *N. ceranae* but proved to be toxic to the infected lepidopteran cells (IPL-LD 65Y) used in the study^19^. In the absence of efficient agents for the treatment of nosemosis, various substances, e.g., essential oils and plant extracts, are being intensely investigated^15^.

Porphyrins are a group of biologically important molecules that are used as anticancer drugs, especially in photodynamic therapy^20,21^. In cells, the iron protoporphyrin IX (PPIX) complex is essential to the function of a number of proteins where it binds to polypeptide chains, e.g., haemoglobin, myoglobin, cytochrome *c*, peroxidase, and catalase. However, considering the applications of porphyrin derivatives, they have considerable hydrophobicity, limiting their applicability in medical purposes and other applications. For example, PPIX aggregates in water into supramolecular assemblies, decreasing its solubility^22^.

**Figure 1:**
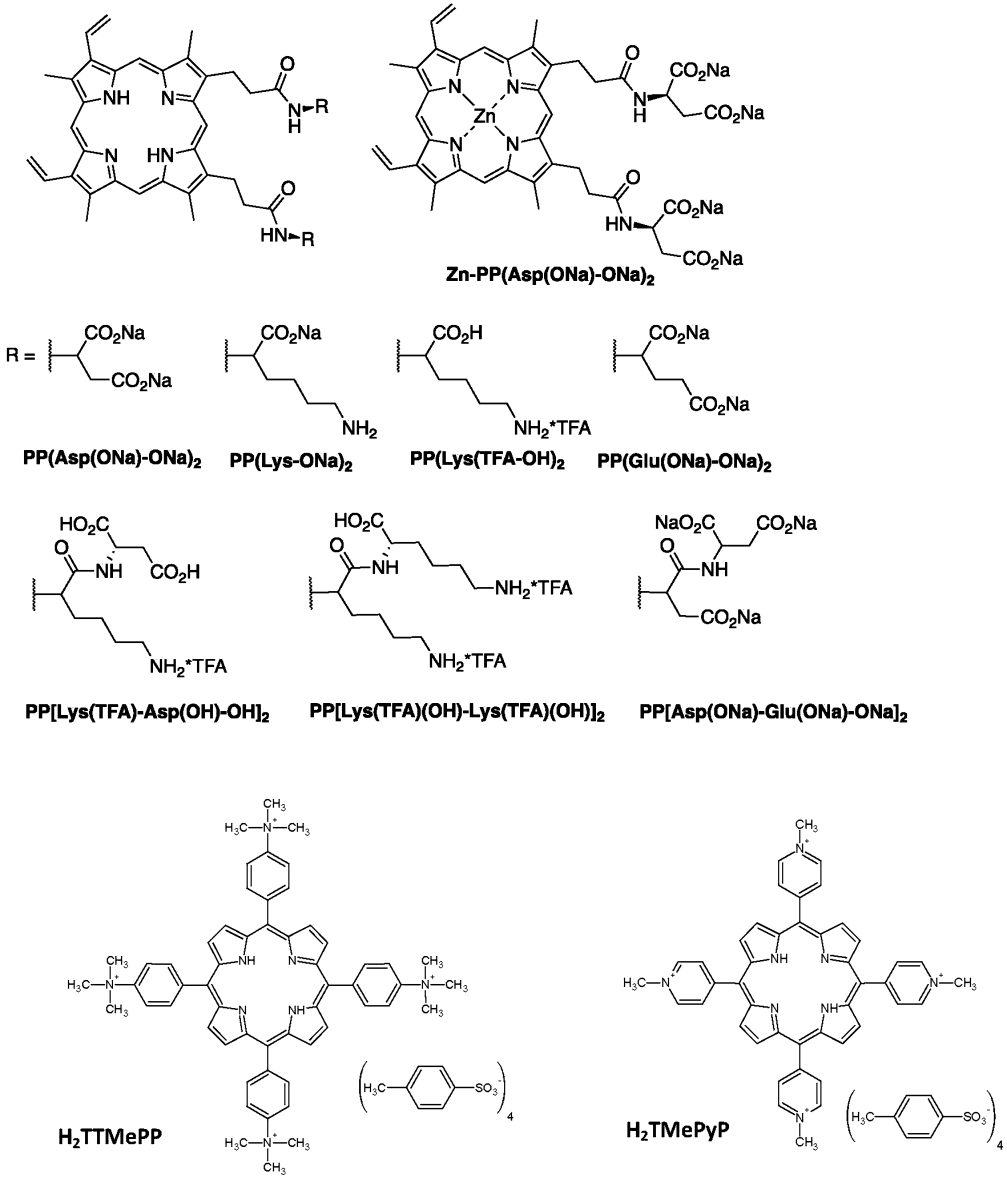
Molecular structures of the porphyrinoids used in the study.

In a previous study, we showed that PPIX conjugated to aspartate moieties showed good water solubility and, importantly, reduced the infectious ability of *N. ceranae* spores and prevented spore development in honeybees^23^. The present work represents an extension of our previous research. We have conducted in vitro investigations of protoporphyrin amide derivatives with other amino acid moieties as active agents against *Nosema* spp. spores relative to the activities of two cationic porphyrins, TMePyP and TTMePP (Fig. 1). In particular, we examined the bioactivities of the porphyrins to elucidate their mechanism of action and showed that some protoporphyrin amides display high antimicrosporidian activity and may represent promising bioactive compounds for combating nosemosis in honeybees. The biological effects of the selected porphyrins against microsporidian spores were proved in an in vivo experiment using caged honeybees (*A. mellifera*).

## Results

### Determination of *Nosema* infection level and spore viability

**Figure 2:**
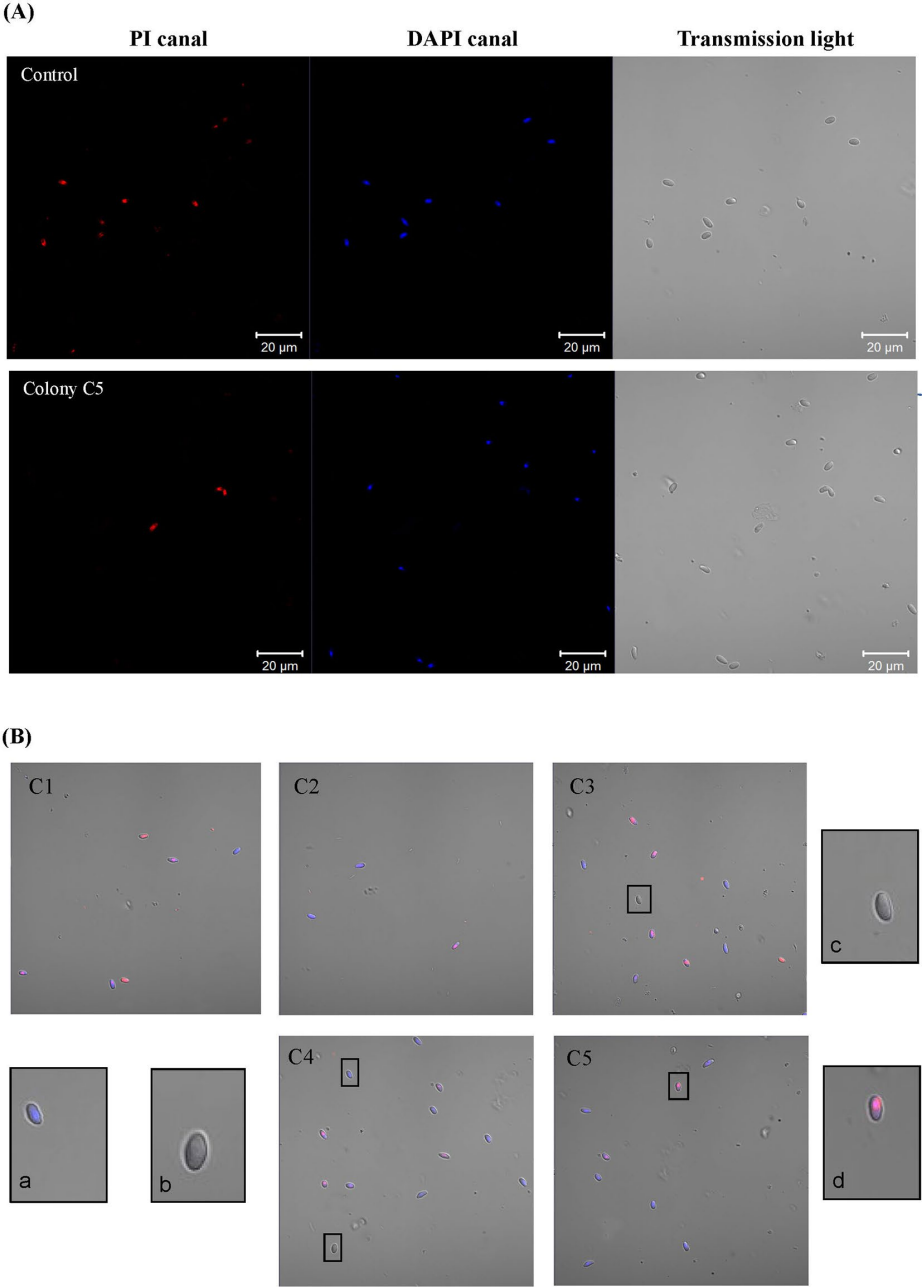
Confocal microscopy images showing dead (red ovals) and live *Nosema ceranae* spores stained with PI (**A**) and *N. ceranae* spores isolated from different winter beehive debris double-stained with DAPI and PI at ×100 (**B**). Live spores—not stained by PI (a); extruded spores—not stained by PI and DAPI (b and c); dead spores—stained by PI and DAPI (d). C1-C5 correspond to the selected honeybee colonies.

In the initial stage of this work, the number of *Nosema* spp. spores in dead bees collected from winter beehive debris was estimated. Nosemosis was confirmed in 20 of the 26 analysed bee colonies (Table S2). The five colonies with the highest infection rates were nos. 54, 60, 70, 77 and 78, which were referred to as C1, C2, C3, C4, and C5 and showed 2.22, 1.58, 2.50, 6.37, and 2.88 × 10^8^ spores per bee, respectively (Table 1). Colony C1 was removed from further testing due to the abundance of yeast cells. As the next step in this study, the viabilities of the spores (expressed as a percentage of live cells) from the four other colonies were analysed before being treated in vitro with porphyrin. Figure 2 shows the confocal microscopy images of the spores. The *Nosema* spores appeared as blue ovals as a result of DAPI uptake by both live and dead cells, and the extruded spores remained colourless (Fig. 2b). The percent values of dead cells in the isolated spores stained with PI (bright red ovals) are presented in Table 1. Among the colonies evaluated, the lowest proportions of dead cells (9.1% and 9.6%) were detected in the spore suspensions from colonies C2 and C5, whereas the highest (52%) was detected from colony C3. The control sample containing heat-inactivated microsporidia showed up to 97.2% dead spores (Fig. 2A).

**Table 1:** Number of *Nosema ceranae* spores in different winter beehive debris and determination of *Nosema* spore viability. Control – spores from colony C3 inactivated with heat at 80 °C for 1.5 h.

Colony	Number of *Nosema* spores per bee (×10^6^)	Dead spores (%)	Live spores (%)
Control	250	97.2	2.8
C1	220	100	0
C2	158.4	9.1	90.9
C3	250	52	48
C4	636.8	32.5	67.5
C5	288	9.6	90.4

### The effect of the selected porphyrins on *Nosema ceranae* spore number and viability

**Figure 3:**
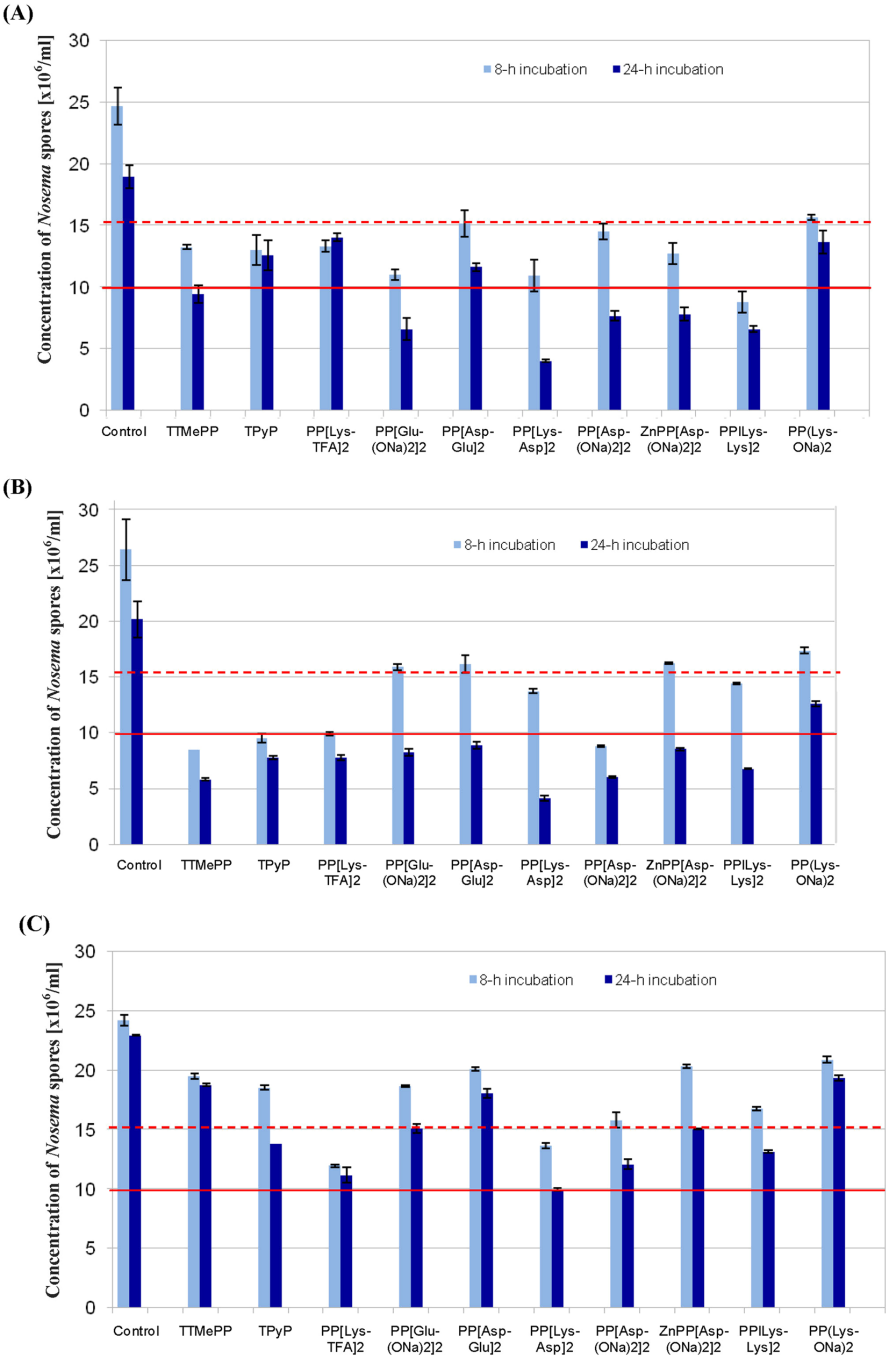
The effects of the tested porphyrinoids on the number of *Nosema ceranae* spores in the dark at porphyrin concentrations of 100 µM (**A**), 50 µM (**B**), and 10 µM (**C**). Control—spores incubated without porphyrinoids.

Suspensions of *N. ceranae* spores were incubated in the dark in the presence of different porphyrins at concentrations of 10, 50 and 100 µM. Compared to that in the control group, the spore number in all the experimental groups was significantly reduced (Fig. 3A–C). Porphyrins, especially PP[Lys-Asp]_2_, PP[Lys-TFA]_2_ and PP[Asp(ONa)_2_]_2_, significantly (*p* < 0.0001) reduced the number of *Nosema* spores at concentrations of 10 µM and showed better activity than that of commercial TMePyP and TTMePP. After 8 h of incubation, 43.6%, 50.6%, and 34.7% reductions were observed for PP[Lys-Asp]_2_, PP[Lys-TFA]_2_ and PP[Asp(ONa)_2_]_2_, respectively, and the reductions reached 56.6%, 51.3% and 47.3% after 24 h (Fig. 3C). At 50 µM, all the porphyrins significantly reduced the number of microsporidia after 8 h and 24 h of incubation (F_(10, 517)_ = 41.53; *p* < 0.001 and F_(10, 517)_ = 41.66; *p* < 0.01, respectively). After 24 h of incubation, PP[Lys-TFA]_2_, PP[Lys-Lys]_2_, PP[Lys-Asp]_2_ and PP[Asp(ONa)_2_]_2_ reduced the number of spores by 61.5%, 66.8%, 79.6% and 70%, respectively. Lower reductions in spore counts were observed for PP[Lys-ONa]_2_ (37.5%), PP[Glu(ONa)_2_]_2_ (59.1%), and ZnPP[Asp(ONa)_2_]_2_ (57.7%). The commercial porphyrins TTMePP and TPyP reduced the numbers of spores by 71.1% and 61.3%, respectively (Fig. 3B). When the concentration of the porphyrins was increased to 100 µM, only in the case of PP[Glu(ONa)_2_]_2_ were the reductions in spore counts (65.1%) observed after 8 (p < 0.01) and 24 h (*p* < 0.05) of incubation greater than those achieved by the same compound at 50 µM (Fig. 3A). Most of the porphyrins showed a decrease in activity after 24 h of action (H_(10, 517)_ = 41.09; *p* < 0.001). A slight increase in the reduction in the number of spores was also observed for PP[Lys-Lys]_2_ (64.3%) and ZnPP[Asp(ONa)_2_]_2_ (48.3%) after 8 h of incubation; however, after 24 h of incubation, the effect remained unchanged in relation to that at 50 µM. The efficacy of most porphyrins, especially TTMePP (50.1%), TPyP (33.5%), and PP[Lys-TFA]_2_ (25.9%), decreased after 24 h of action.

**Figure 4:**
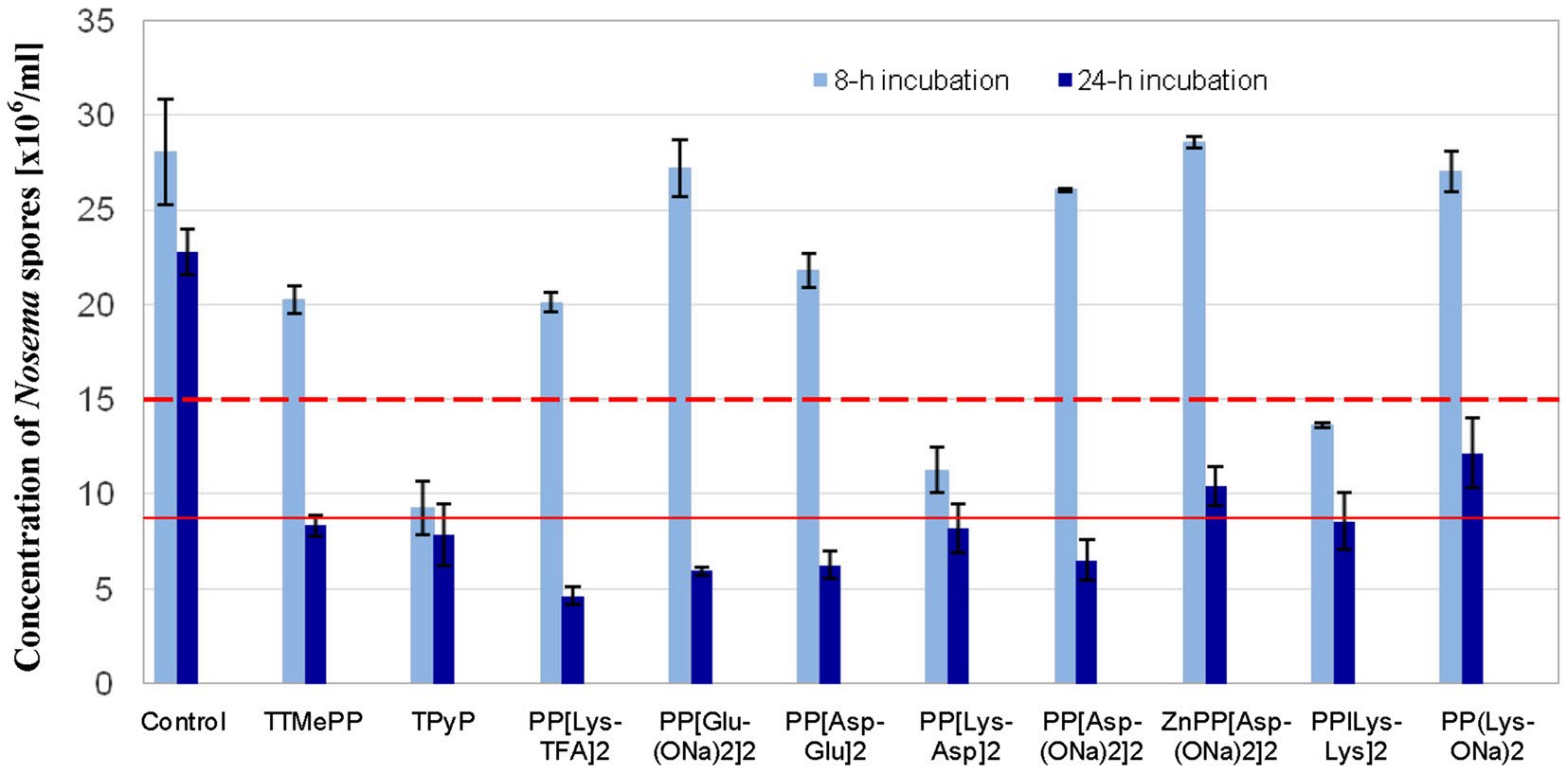
The effects of porphyrinoids on the number of *Nosema ceranae* spores under light irradiation. Porphyrin concentration: 50 µM. Control—spores incubated without porphyrinoids.

Generally, no substantial enhancement in the porphyrin activities was observed during incubation in the light relative to during incubation in the dark (Fig. 4). A higher reduction in the spore number after 24 h of incubation was only found for PP[Lys-TFA]_2_ (79.7%), PP[Glu(ONa)_2_]_2_ (73.9%), PP(Asp-Glu)_2_ (72.5%) and PP[Lys-ONa]_2_ (46.5%). However, after a shorter incubation time (8 h), the efficacies of all the porphyrins became drastically lower than those achieved during incubation in the dark (H_(10, 517)_ = 37.93; *p* < 0.0001), except for the porphyrins TPyP, PP[Lys-Asp]_2_ and PP[Lys-Lys]_2,_ for which the magnitudes of the reductions in the number of *Nosema* spores slightly increased (up to 67.0%, 59.8%, and 51.3%, respectively).

**Figure 5:**
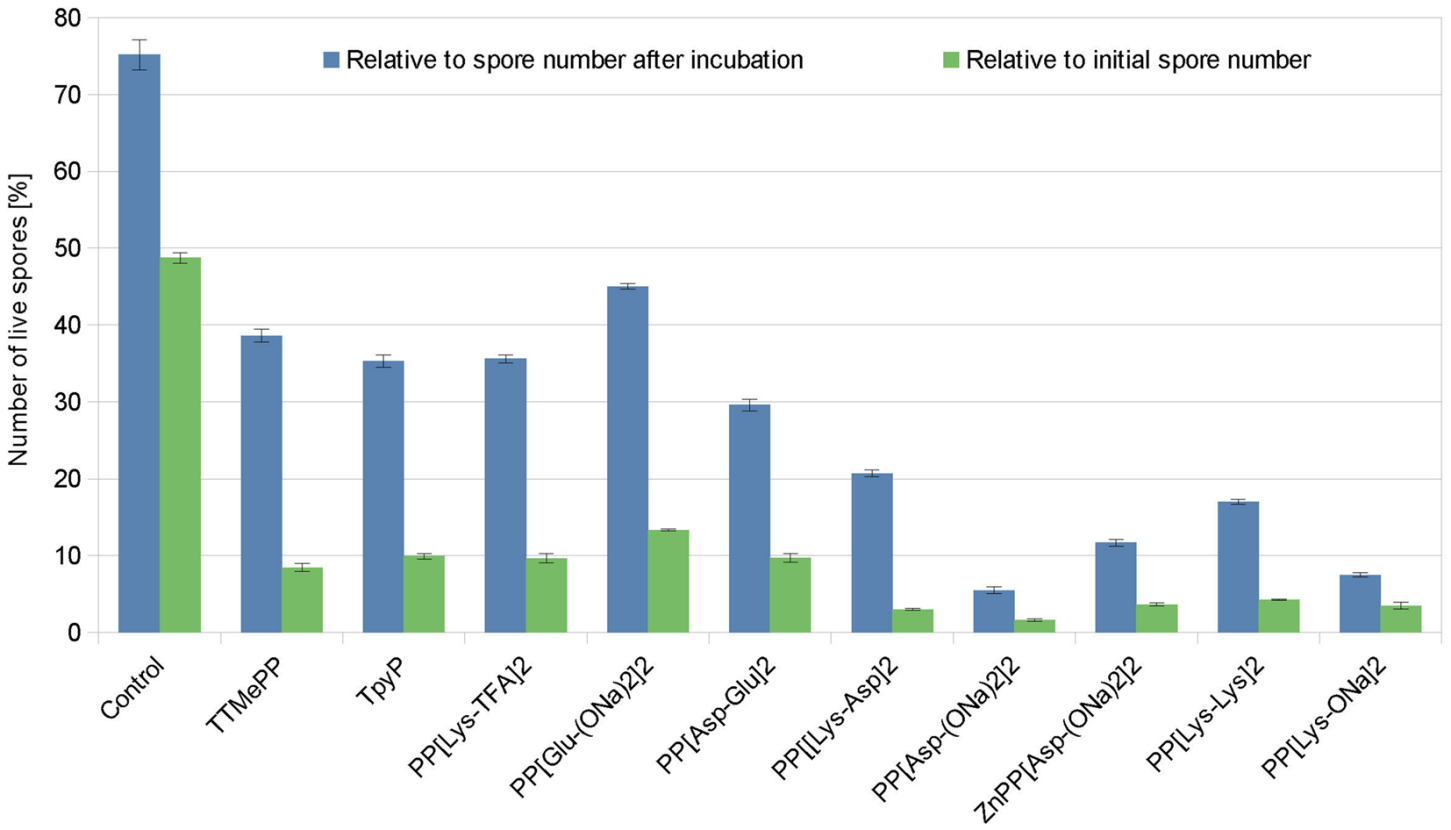
Viability of undamaged *N. ceranae* spores, which remained undamaged after 24 h of treatment with porphyrins at 50 µM.

Live-cell confocal imaging of the treated spores, which remained undamaged after 24 h of incubation with the porphyrins at 50 µM, showed that among the porphyrins evaluated, the smallest percentages of cells survived after treatment with PP[Asp(ONa)_2_]_2_ (5.5%), PP[Lys-ONa]_2_ (7.5%) and ZnPP[Asp(ONa)_2_]_2_ (11.7%). Relative to the number of spores remaining after incubation and the total number of cells before incubation (with 90.4% of live spores), 75.2% and ~ 50% of the untreated control spores, respectively, were alive. However, the prevalence of live cells after treatment with the porphyrins in relation to the initial number of spores (3 × 10^7^ spores/mL) did not exceed 13.3% but was as low as 1.6% for PP[Asp(ONa)_2_]_2_, 3.0% for PP[Lys-Asp]_2_, 3.5% for PP[Lys-ONa]_2_, 3.65% for ZnPP[Asp(ONa)_2_]_2_ and 4.3% for PP[Lys-Lys]_2_ (Fig. 5).

### Porphyrin uptake by *Nosema ceranae* spores

To further ascertain the mechanism of porphyrin incorporation into the cell (active transport or passive diffusion), we analysed cellular uptake by freshly harvested spores (live spores) and heat-inactivated spores (dead spores). The uptake of PP[Lys-TFA]_2_ and PP[Lys-Lys]_2_ by the spores was studied to understand the differences in the activities of these porphyrins, which bear single and double amino acid moieties (dipeptide), respectively.

**Figure 6:**
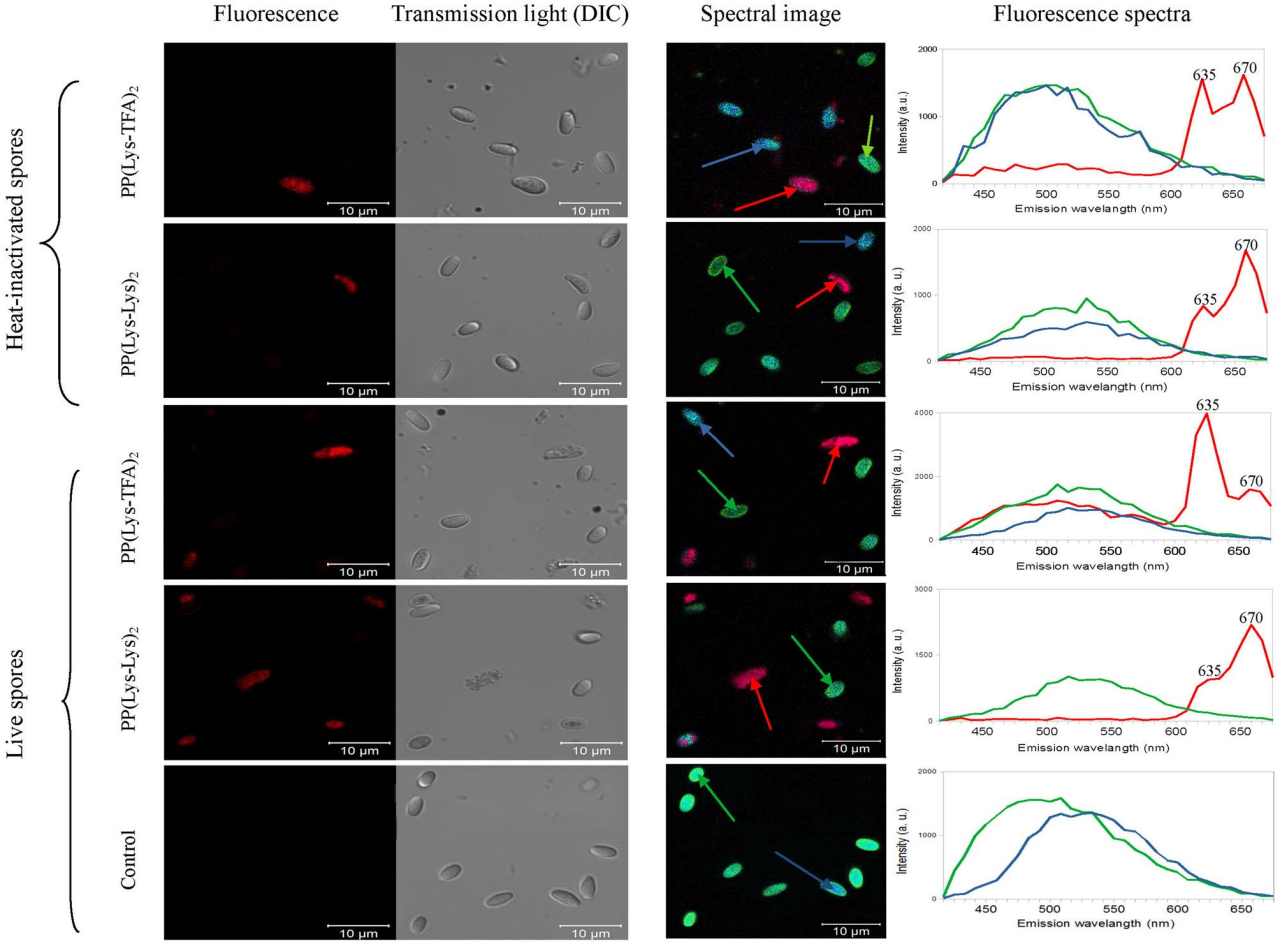
Confocal fluorescence microscopy images of *N*. *ceranae* cells (left) after 4 h of treatment with the porphyrins, PP[Lys-TFA]_2_ or PP[Lys-Lys]_2_, and the corresponding fluorescence spectra (right). Pictures of the heat-inactivated and fresh (live) spores are shown. DIC—differential interference contrast. Red fluorescence indicates the presence of porphyrin inside the cell. Fluorescence spectra were collected from the points indicated by arrows. Control—spores not treated with porphyrin.

**Figure 7:**
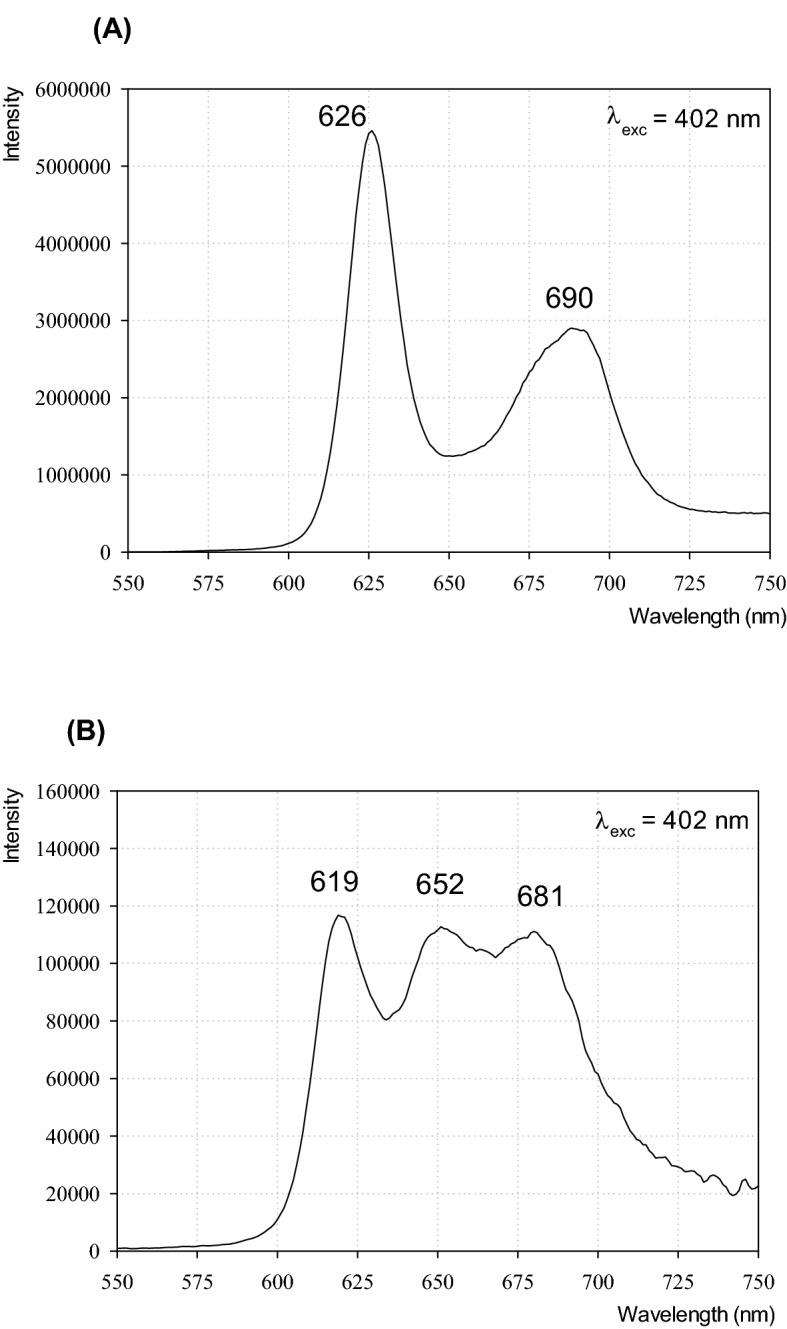
The fluorescence spectra of PP[Lys-TFA]_2_ (**A**) and PP[Lys-Lys]_2_ (**B**) in 0.5% saccharose aqueous solution. The excitation wavelength λ_exc_ = 402 nm. Porphyrin concentration—50 µM.

Porphyrin uptake by microsporidia with different levels of viability was determined by porphyrin fluorescence measurements using confocal microscopy (Fig. 6). The spores were excited with a laser at 405 nm after 2-h and 4-h incubation with the porphyrins, and images were collected using confocal microscopy with two approaches: fluorescence detection in the red light range (Fig. 6, left panel) and a spectral imaging detector (Fig. 6, middle and right panels). The cells containing accumulated porphyrins emitted red fluorescence with emission spectra characteristic of the porphyrin, namely, two characteristic maxima at 635 nm and 670 nm (Fig. 6, right panel). However, the intensities of the maxima were different for the two porphyrins used. For PP[Lys-TFA]_2_, the more intense band was at 635 nm, whereas for PP[Lys-Lys]_2_, the band at 670 nm was dominant. Importantly, these results are consistent with the emission spectra of the porphyrins recorded in a 0.5% sucrose solution (Fig. 7), indicating that the red light fluorescence detected from the spores can be attributed to the porphyrins in the free state. The fluorescence spectra of PP[Lys-TFA]_2_] and PP[Lys-Lys]_2_ in 0.5% sucrose, each with two well-resolved emission peaks, are presented in Fig. 7.

Neither of the porphyrins showed cell wall localization, and the porphyrin fluorescence signal was predominantly from the intracellular area. Interestingly, in the spores subjected to a high temperature, the porphyrins did not accumulate (lack of red fluorescence), and the majority of the spores displayed only autofluorescence in a broad range of their fluorescence spectra (420–630 nm). When live spores were incubated with PP[Lys-TFA]_2_ and PP[Lys-Lys]_2_ for 4 h, red fluorescence was observed in 32.4% and 54.5% of the cells, respectively (Table 2). In the case of heat-inactivated cells (containing 2.8% live spores), only 10.5% and 5.6% of the cells showed red fluorescence with emission spectra characteristic of the corresponding porphyrins (Table 2).

**Table 2:** The relationship between the porphyrin uptake by microsporidia and *Nosema* spore viability.

Porphyrin	Incubation time (h)	Mean (%) spores showing red fluorescence (± SD)	
		A—heat-inactivated spores (2.8% of live spores)	B—fresh spores (90.4% of live spores)
PP[Lys-TFA]_2_	2	0 (± 0.00)^a^	27.3 (± 0.7)^b^
	4	10.5 (± 0.45)^a^	32.4 (± 0.68)^b^
PP[Lys-Lys]_2_	2	0 (± 0.00)^a^	30.2 (± 0.89)^b^
	4	5.6 (± 0.62)^a^	54.5 (± 0.81)^c^

The values are expressed as the means ± standard deviations (SD). Lowercase letters (a, b, and c) indicate statistically significant differences between groups A and B.

The percentage of spores incorporating the porphyrin was determined by dividing the number of spores stained with porphyrin (red) by the number of spores counted using the microscope’s bright field. Lowercase letters (a, b, c, and d) indicate statistically significant differences between groups A and B (F_(7, 120)_ = 55.63; *p* < 0.001) (one way ANOVA, Tukey’s test). The post hoc comparison showed no significant differences in the number of heat-inactivated spores between the two porphyrins (*p* > 0.05). However, statistically significant differences between the two porphyrins were observed for fresh spores after 4-h incubation (*p* < 0.001).

### Effect of in vivo porphyrin treatment on the course of nosemosis and mortality of honeybees

**Figure 8:**
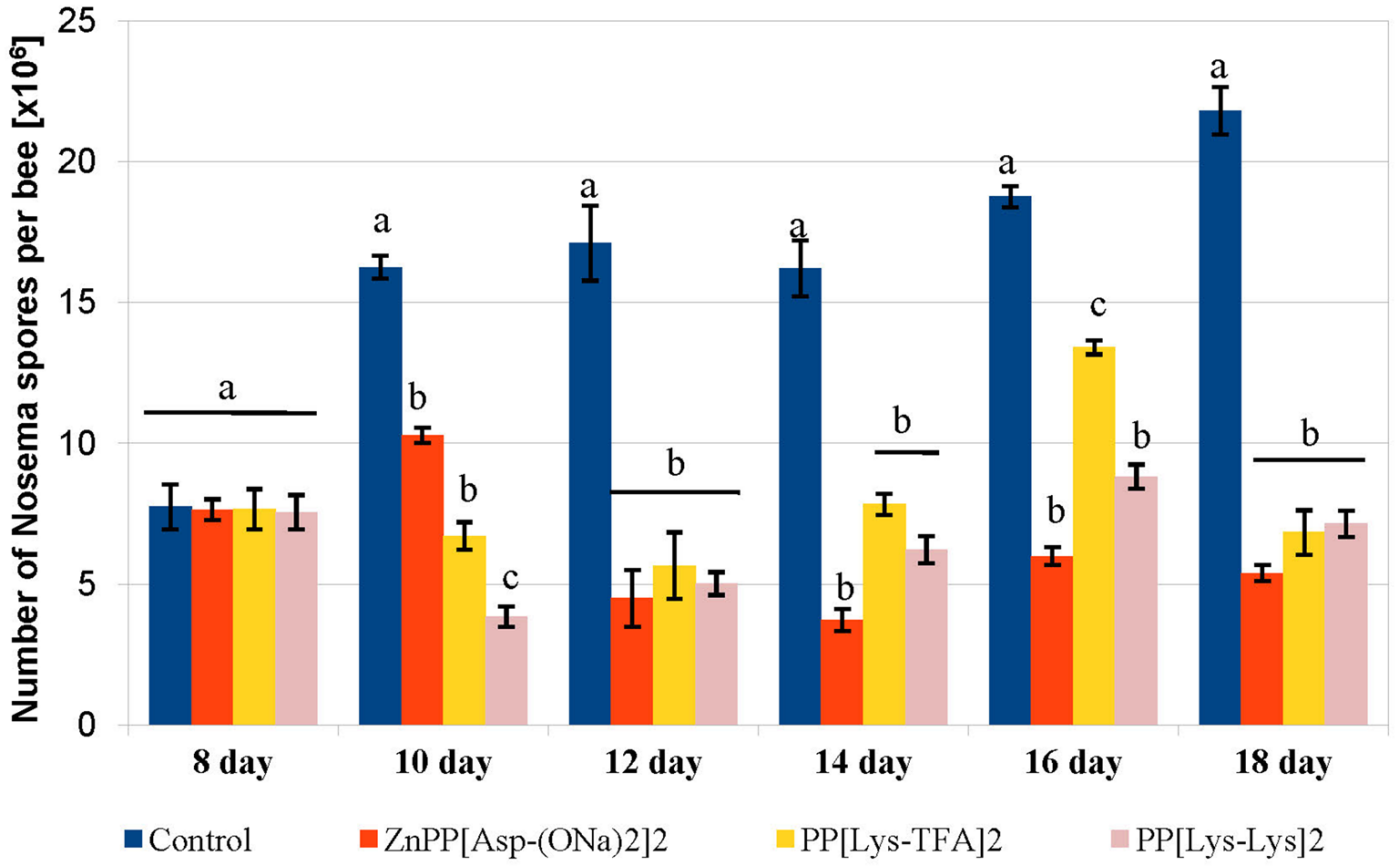
In vivo effect of protoporphyrin IX derivatives on the number of *Nosema ceranae* spores in infected *Apis mellifera*. Statistically significant differences between the control group and treated groups within a particular day are indicated with lowercase letters: a, b, c; error bars represent standard errors of data.

**Figure 9:**
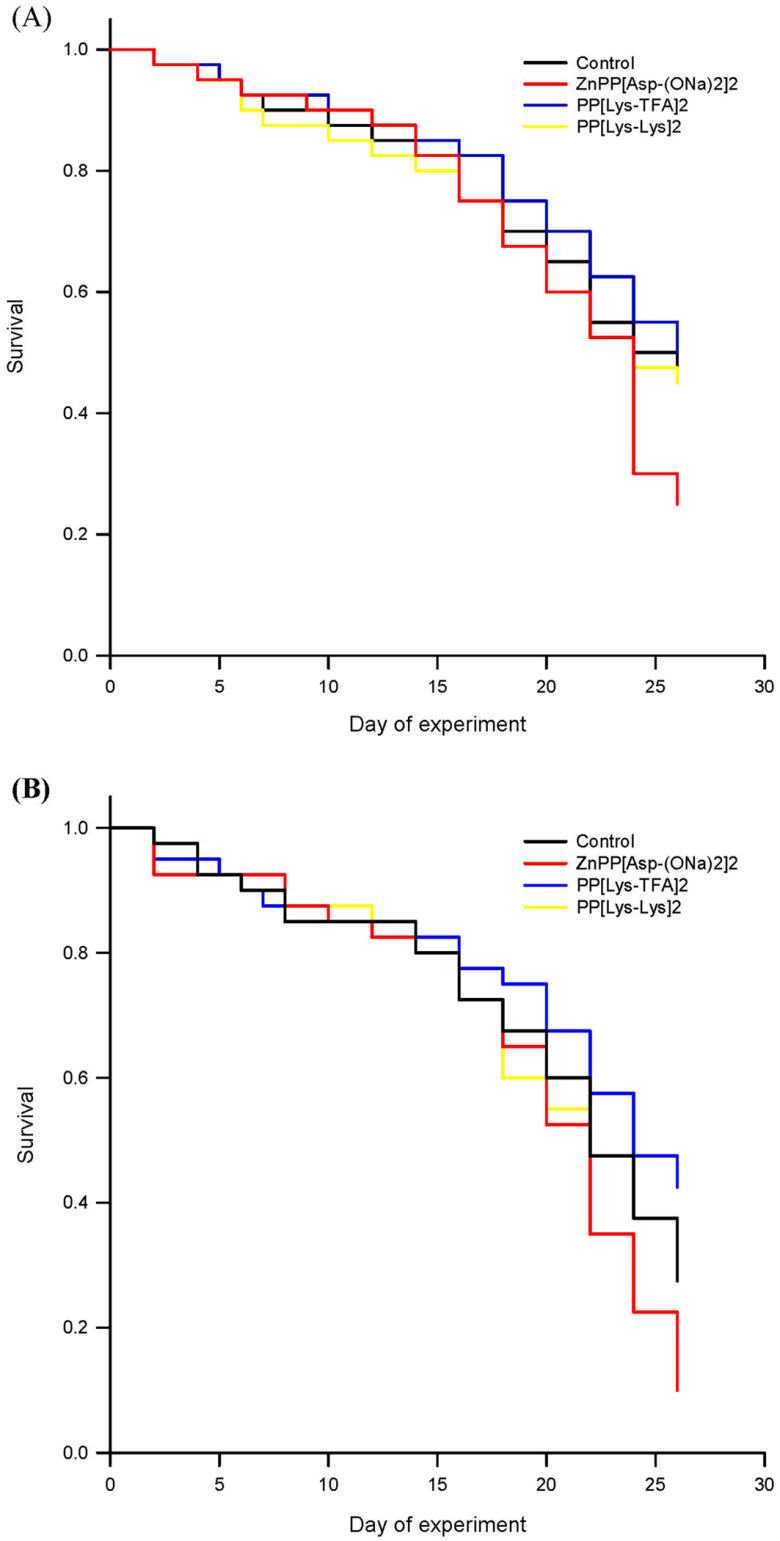
Kaplan–Meier survival curves for (**A**) uninfected and (**B**) *N. ceranae-*infected honeybees treated with selected protoporphyrin IX derivatives in the sucrose syrup supplement.

To verify in vivo activity of porphyrins, the biological effects of the selected porphyrins on the *N. ceranae* development was investigated in honeybee hosts using cage experiments (Fig. 8). In addition, mortality of both healthy and *Nosema*-infected honeybees was examined (Fig. 9). From day 10, each of the porphyrins, PP[Lys-TFA]_2_, PP[Lys-Lys]_2_, and ZnPP[Asp(ONa)_2_]_2_, administered to the infected honeybees in a sucrose syrup supplement (at a concentration of 100 μM) significantly decreased the spore counts, compared to the honeybees in the control group, which were exposed to pure sugar syrup only (F_(3, 76)_ = 123.21; *p* < 0.001). On day 18, the spore load was reduced by 69–76% in the subjects treated with the porphyrins compared to the control honeybees (F_(3, 76)_ = 143.88; *p* < 0.001), which were not treated with the compounds (Fig. 8); however, the differences between the porphyrin treatments after day 18 were not significant. Statistically significant differences between PP[Lys-Lys]_2_ and PP[Lys-TFA]_2_ were observed on days 10 and 16, and the former showed better activity in the reduction of the spore load than the later compound (*p* < 0.001; *p* < 0.001). No *Nosem*a spores were detected in the negative control (uninfected) bees during the 18 days of the experiment.

Among *Nosema*-infected honeybees, we did not observe an increase in the lifespan of the honeybees treated with the porphyrin solution compared to the control honeybees (*p* = 0.054). Lower mortality (57%) was noted in the PP[Lys-TFA]_2_-treated honeybees over a period of 26 days, although it was not significantly different from the mortality of the control group of infected honeybees (72%) (Fig. 9B). In turn, the mortality rates in the uninfected bees treated with porphyrins were similar for PP[Lys-TFA]_2_ and PP[Lys-Lys]_2_, and these did not differ significantly from the mortality of negative control bees (53%) (Fig. 9A). Moreover, both healthy and infected honeybees exposed to ZnPP[Asp(ONa)_2_]_2_ showed higher mortality (76%), indicating that this metalloporphyrin may be toxic to honeybees.

## Discussion

Microsporidia are parasites spread by intracellular germination, and their propagation occurs exclusively in infected host cells. The only cell culture system for *Nosema* is based on a lepidopteran cell line susceptible to infection by both *N. ceranae* and *N. apis*^24^. Although this system enabled to illustrate the entire life cycle of these microsporidia, it is not commonly used for the propagation of microsporidia for screening candidate compounds as new therapeutic agents for the anti-*Nosema* treatment^24^. Therefore, compared to studies on other genera of microsporidia^25,26^, reports on in vitro studies of potent compounds that affect *Nosema* spp. are limited^19^. In the present work, an in vitro procedure was developed to screen the abilities of different porphyrins to inactivate microsporidian cells. First, we selected dead bees from winter hive debris of the five colonies that had the highest number of *Nosema* spores per bee (Table 1). The *Nosema* spores were then selected based on their viability determined using a combination of DAPI and PI staining (Table 1 and Fig. 2)^27–29^. Fortunately, PI-stained spores emit strong visible fluorescence with a relatively long lifetime (> 10 s)^30^. This two-step experimental procedure facilitated the selection of microsporidia with the lowest proportion of dead spores (9.1% and 9.6% dead cells) (Fig. 2B), which was important, as microsporidia with diminished viability are less able to infect honeybee’s intestines^31^. The measurements were referenced to a negative control containing heat-inactivated spores. Heating *N. apis* spores for at least 15 min at 60 °C completely kills the parasite^32^. *N. ceranae* is more resistant to higher temperatures and has higher biotic potential than its congener^33,34^. Therefore, heat treatment at 80 °C for 1.5 h was used^27^, and as expected, this procedure killed almost all of the *N. ceranae* spores (97.2% dead spores). Importantly, the heat-treated microsporidia cells remained intact, making them useful and appropriate controls for subsequent experiments.

Herein, we examined the effects of various protoporphyrin amide derivatives and two commercial porphyrins on *Nosema* microsporidia to characterize the influence of the identity of amino acid moieties on the porphyrin activity to provide insight into their mechanism of action on microsporidia. In all cases, significant reductions in the number of spores compared to the control were observed (Fig. 3). The porphyrins PP[Lys-Asp]_2_, PP[Lys-TFA]_2_ and PP[Asp(ONa)_2_]_2_ exerted potent activities at concentrations as low as 10 µM, showing distinctly greater effectiveness than the cationic porphyrins TMePyP and TTMePP. However, *N. ceranae* was more susceptible to damage in 50 µM porphyrin solutions than in 10 and 100 µM porphyrin solutions. The efficacies of most porphyrins at the highest concentration (100 µM) dropped during the 24 h evaluation period. Presumably, self-aggregation of the porphyrins was responsible for the decrease in activity. Self-aggregation of porphyrinoids in aqueous media is common and is mainly driven by π–π stacking interactions between the macrocycles, leading to the formation of dimers, trimers, oligomers, and/or large-scale aggregates. PPIX is particularly prone to aggregation in aqueous environments via intramolecular interactions between the hydrophilic COOH group of the propionic acid and the hydrophobic porphyrin core^22^. In most of the 100-µM samples, we found significant amounts of precipitated red pellets after 24 h of incubation (data not shown), and we assume that the lower activity was caused by the formation of higher-order aggregated porphyrin species that can further bind to *Nosema* cells, leading to porphyrin stacking. Such a phenomenon was not observed for PP[Glu(ONa)_2_]_2_ or PP[Lys-Lys]_2_, which, together with the fact that only these porphyrins showed stronger antimicrosporidial effects at 100 µM than at 50 or 10 µM (Fig. 3A), supports this hypothesis.

The bioactivity of the porphyrins against *Nosema* was drastically altered by structural alterations to the porphyrin periphery (Figs. 1, 3A–C). Depending on the amino acid moieties and the length of these side chains on the PPIX molecule, the magnitudes of the reduction in the number of spores relative to the control varied. PPIX derivatives conjugated to aspartate or lysine exerted higher activities than PP[Glu(ONa)_2_]_2_ bearing glutamate moieties. An approximately twofold higher concentration of PP[Glu(ONa)_2_]_2_ was needed to produce a destructive effect similar to that observed with PP[Asp(ONa)_2_]_2_ (Fig. 3A,B). By comparing PP[Lys-ONa]_2_ and PP[Lys-TFA]_2_, we found that the type of counterion for the amino acid functional group is of great importance for the antimicrosporidian activity (Fig. 3B,C). Higher activities can also be achieved with porphyrins having two amino acid moieties, as seen with PP[Lys-Lys]_2_ and PP[Lys-Asp]_2_, than those having one amino acid moiety (Fig. 3), probably due to their greater ability to penetrate cell walls and membranes. l-Amino acids were incorporated into the adducts as linkers to improve porphyrin uptake and increase potency, and porphyrins with longer peptide chains were more effective than porphyrins bearing a single amino acid moiety^35–37^. The exceptionally low bioactivity of PP[Asp-Glu]_2_ in comparison with derivatives of PPIX bearing a single amino acid moiety can be explained by the presence of Glu at the periphery of the molecule, and this behaviour is consistent with the relatively low reduction in spore number observed for PP[Glu(ONa)_2_]_2_. We also demonstrated that the presence of a metal ion in the porphyrin ring influences porphyrin bioactivity. In our study, the insertion of Zn(II) into the porphyrin ring decreased the activity of PP[Asp(ONa)_2_]_2_ (Fig. 3B and C). This effect is probably due to the strong tendency of zinc-coordinated porphyrins to stack as a result of the changes in the electron-donating properties of the central cavity of the metalloporphyrin ring^38^. Thus, the interactions of the metallated porphyrins with endogenous compounds in the cells may be limited.

Non-metallated porphyrins are known to be much more effective against microorganisms when irradiated than when kept in the dark^39^. In particular, cationic porphyrins are able to induce photosensitized damage to DNA via strand scission by producing reactive oxygen species, e.g., ^1^O_2_, upon irradiation^36,40,41^. The effect of porphyrin irradiation on *Nosema* spore inactivation was analysed in this work. None of the porphyrins more efficiently deactivated the spores under irradiation than they did in the dark (Fig. 4). Interestingly, after 8 h of incubation, the activities of most porphyrins dropped drastically, most likely due to partial photodegradation of the porphyrin molecules. These results further confirmed that the inactivation of microsporidia is not associated with a photodynamic mechanism. They are consistent with our previous findings, which revealed that microsporidia treated with metal-free porphyrins were as sensitive in the presence of light as they were in the absence of light and equally lost their ability to infect honeybees^23^.

Since porphyrins induce morphological changes in the spore cell wall with the appearance of deformities in the exosporium layer^23^, the porphyrin targets are likely localized on the surface of or in the *N. ceranae* cell wall. The affinity of porphyrins for external structures, namely, the cell walls and cytoplasmic membranes, has been reported for bacteria^42^ and yeasts^43^. In the next step of this study, using fluorescence confocal microscopy, we assessed whether the porphyrins PP[Lys-TFA]_2_ and PP[Lys-Lys]_2_ are localized in the spore wall (within the endo- and exosporium) or whether they enter the cell, thus accessing internal targets. As demonstrated, the red light fluorescence attributed to the porphyrins in the free state was evenly distributed across the cells, indicating that the porphyrins crossed intact cell walls and were not localized exclusively in one compartment, as the spores were homogenously saturated (Fig. 6). This finding allows the possibility that the porphyrins could interact with numerous intracellular components in the sporoplasm (for example, the nucleus, proteins or polar tube), increasing the toxicity of the compound and leading to a decrease in the viability of the spores. PP[Lys-Lys]_2_ showed a higher degree of accumulation in microsporidia than PP[Lys-TFA]_2_ (Table 1), probably because the former porphyrin is better able to permeate the membrane. Therefore, the increased sensitivity of *N. ceranae* to amphiphilic amide derivatives of PPIX could be attributed to the increased permeability of the two layers of the microsporidian spore wall to protoporphyrins bearing peptides of two (or more) amino acids. Increased uptake is greatly desired, as it often leads to greater efficacy. The water-soluble amphiphilic and cationic porphyrins are able to permeate cell walls and membranes of yeast and exhibit more pronounced photoactivity^43^. Compared to neutral (zwitterionic) porphyrins or porphyrins with non-charged head groups (e.g., ethylene glycol-based groups), cationic porphyrin dyes have a higher affinity for plasma membranes^44^. Since the studied cationic porphyrins (without amine functional groups) are also active against *Nosema* spores, the number and position of the charges, as well as the optimal ratio between hydrophilic and hydrophobic regions, should be considered when designing an ideal porphyrin-based antimicrosporidial agent; hydrophilicity facilitates distribution, and lipophilicity facilitates better cellular uptake^45^.

Importantly, we revealed that in contrast to dead spores, in which porphyrin accumulation occurred only to a very small extent, live spores did accumulate porphyrin (Table 2). Our findings indicate that live cells incorporate porphyrins through uncompromised membranes. Although the details of the porphyrin uptake mechanism remain unclear, we may assume that an active transport pathway exists and that this pathway facilitates the uptake of porphyrins into the microsporidia interior. Our data suggest that passive diffusion through the spore wall is unlikely to overcome the barrier presented by the electron-dense outer exospore layer and electron-lucent inner endospore. To the best of our knowledge, there are no reports describing such a phenomenon in *N. ceranae*. In bacterial pathogens, metalloporphyrins and metal-free porphyrins are known to exploit the haem/Hb uptake mechanism (which provides bacteria with iron)^46^, which is based on the outer membrane receptor HemR transporting haem into the periplasm by an energy-dependent process. Since porphyrin-based reactivity in microsporidia cells is completely unknown, the mechanism of *Nosema* spore destruction by porphyrins remains unclear. In addition, very little is known about the mechanism of porphyrin bioactivity in the dark, and not much information about their interactions with phospholipid membranes or endogenous compounds at the molecular level is available^20,47,48^. Hemin bound to microbial cells exerts its cytotoxicity by peroxidative and oxidative reactions independent of light^49^. Temizel et al.^47^ observed that oleylamine-conjugated porphyrins form effective interactions with cell membranes and cause apoptosis in cancer cells. PPIX induces apoptosis by binding to the N-terminal domain of p53, leading to activation of the p53 tumour suppressor protein^50^. In general, permeation of the porphyrin photosensitizers through the plasma membrane into cancer and microbial cells leads to cell inactivation via events including impairment of mitochondrial function and inhibition of the synthesis of macromolecular compounds^45,47,51^. Hence, it is possible that porphyrins in microsporidia cell target proteins that induce apoptosis and the disruption of the spore wall.

Regardless of the mechanism of the anti-*Nosema* action of porphyrins, in addition to in vitro activity, the porphyrins PPIX[Lys-TFA]_2_ and PPIX[Lys-Lys]_2_ decreased significantly the number of *Nosema* spores in the infected honeybees when administered in vivo at the 100 µM concentration. Noteworthy, they both had no toxic effect on the honeybees, in contrast to metallated ZnPP[Asp(ONa)_2_]_2_. It should be emphasized that the infected lysine porphyrin-fed bees lived as long as the uninfected control bees. Although zinc-coordinated porphyrin showed high in vitro activity in reduction of the spore number and reduced substantially the spore loads in the infected honeybees, this porphyrin also increased the mortality of both uninfected and *Nosema*-infected honeybees. Most likely, if the experiment had been conducted for a longer period, bees treated with porphyrin ZnPP[Asp(ONa)_2_]_2_ would have died first. Probably, the zinc ion (in its excessive amount) is responsible for the adverse effect on honeybees, most likely by interfering with the ability to generate ROS, leading to disruption of physiological processes and abnormal protein synthesis^52,53^. The metalloporphyrin may also have damaged the intestinal function, contributing to the increase in the mortality and the decrease in the feed intake observed in *Nosema*-infected honeybees (Fig. S4).

These results indicate that selected porphyrins modified appropriately can be used for designing drugs against *Nosema* infections in honeybees. Any differences observed between the in vitro and in vivo activity of the porphyrins against *Nosema* were probably associated with the more complex environment of the bee’s midgut compared to the sucrose solution in which porphyrins were able to directly inactivate spores through the structure–activity relationship. In the honeybees, porphyrins are not only involved in the direct action on the spores but can also interact with epithelial host cells affecting, for instance, immune defense responses to *Nosema* infection.

## Materials and methods

### Chemicals

Amphiphilic protoporphyrin IX amides, **PP[Asp(ONa)-ONa]**_**2**_, **PP(Lys-ONa)**_**2**_, **PP[Lys(TFA)-OH)]**_**2**_, **PP[Glu(ONa)-ONa]**_**2**_, and **PP[Asp(ONa)-Glu(ONa)-ONa]**_**2**_, were synthesized from protoporphyrin IX as described by Maximova et al.^54^ and fully characterized. The metalloporphyrin **Zn-PP[Asp(ONa)-ONa]**_**2**_ was prepared from protoporphyrin IX. The porphyrins with two amino acid moieties as hydrophilic head groups, **PP[Lys(TFA)-Asp(OH)-OH]**_**2**_ and **PP[Lys(TFA)-Lys(TFA)-OH]**_**2**_, were synthesized using a solid-phase technique.

Synthesis and analytical data (*e.g.* ^1^H NMR and ^13^C NMR spectra) of the porphyrins: Zn-PP[Asp(ONa)-ONa]_2_, PP[Lys(TFA)-Asp(OH)-OH]_2_ and PP[Lys(TFA)-Lys(TFA)-OH]_2_ are provided in Supplementary materials S1.

The purity of the porphyrins was ≥ 95%. 5,10,15,20-Tetrakis(1-methylpyridinium-4-yl)porphyrin tetra(*p*-toluenesulfonate) (TMePyP) and 5,10,15,20-tetrakis[4-(trimethylammonio)phenyl]-21*H*,23*H*-porphine tetra-*p*-tosylate (TTMePP) were purchased from Sigma-Aldrich. The molecular structures of the porphyrinoids used in the study are presented in Fig. 1.

### Selection of honeybee colonies with the highest number of live *Nosema* spores

To obtain materials for the study, dead bees from the bottom of the hives were collected in March 2017 and analysed for the presence of spores. Winter beehive debris was collected from 26 honeybee colonies of the experimental apiary of the University of Life Sciences in Lublin, Poland. When needed, the dead honeybees were stored at − 20 °C (for a maximum of 21 days) prior to the isolation of *Nosema* spp. spores. To identify the colony with the highest number of spores, ten whole honeybees from each colony were homogenized in 10 mL of sterile distilled water, and the number of *Nosema* spores was determined in accordance with the methodology described by Hornitzky^55^ and Fries et al.^56^. The prepared cell suspensions were placed onto microscope slides, and the quantity of *Nosema* spp. spores was determined in six fields of view using a Thoma chamber and a Nikon ECLIPSE E200 light microscope.

Three independent samples (10 insects for each sample) were prepared from each colony tested (Table S2). The infection rate is expressed as the number of spores per honeybee. The results are reported as the average value of all replicates.

To identify *Nosema* species in the infected honeybees of the selected colonies, a duplex PCR-based assay able to detect the specific 16S rDNA of *N. apis* as well as *N. ceranae* was performed^57,58^ (Fig. S3). The presence of *N. ceranae* DNA was demonstrated by detection of the specific 16S rRNA gene amplicons (218–219 bp) (Fig. S3).

### Isolation of microsporidia and the determination of spore viability

For spore isolation, only colonies with bees that were predominantly infected by *N. ceranae* were used; colonies infected by both *N. apis* and *N. ceranae* were excluded. Spore preparations were prepared exclusively with microsporidia isolated from honeybees shortly after hive debris collection since the freezing and long-term storage of spores outside host cells affect spore viability and infection rate^33,59^. First, the intestines from infected honeybees of each of the selected hives were isolated. To obtain the spore suspension, intestines from 25 honeybees were gently homogenized on ice in 25 mL of sterile H_2_O. The suspensions were filtered through two layers of sterile gauze and a cotton plug, centrifuged at 6500 rpm for 5 min at 4 °C, and decanted. Next, the pellet was resuspended in 10 mL of H_2_O and centrifuged again. These steps were repeated three times.

To determine dead and live *Nosema* spores, differential staining was used according to the methods described by Fenoy et al.^59^, Cambell et al.^60^, Green et al.^25^, Peng et al.^30^ and McGowan et al.^61^ with slight modifications. Two fluorescent compounds, i.e., 4′,6-diamidino-2-phenylindole (DAPI; to stain the nuclei of the spores) and propidium iodide (PI; to stain the nuclei of dead spores), were used in combination to distinguish live spores from dead spores.

Prior to staining, the microsporidia isolated from the *Nosema*-infected insects were purified by centrifugation (13,000 rpm; 30 min) in a Percoll gradient^30,62^ and then centrifugation twice in H_2_O (2000 rpm, 5 min) to remove host tissues. Subsequently, 400 µL of the *Nosema* suspension was rinsed with PBS (in g/L: NaCl—8; Na_2_HPO_4_—1.42; KCl—0.2; and KH_2_PO_4_—0.27) and centrifuged (2000 rpm, 5 min), and the pelleted microsporidia were suspended in staining solution containing DAPI (100 µg/mL) and PI (24 µM). The samples were incubated in the dark for 30 min at 30 °C with gentle shaking using a Roto-Bot rotator (Benchmark). Next, the samples were centrifuged at 2000 rpm for 6 min and rinsed twice with H_2_O. After each rinse, the samples were centrifuged at 2000 rpm for 4 min. Next, the pellet was resuspended in H_2_O and gently mixed. Then, 5-µL aliquots of the suspension were transferred in duplicate onto microscope slides for cell imaging.

The viability of the *N. ceranae* spores was immediately evaluated using an LSM780Zeiss laser scanning confocal microscope equipped with an AxioObserverZ1 inverted microscope and a Plan-Apochromat 63×/1.40 Oil DIC M27 objective. The viability was determined as a percent of live spores. The microscopy analysis was carried out using a 405-nm diode laser for the excitation of DAPI and a 514 nm argon laser for the excitation of PI. Fluorescence emission spectra were recorded in the range of 410–450 and 550–630 nm, respectively. Images in transmitted light mode were simultaneously obtained to differentiate the extruded spores that were not stained by either PI or DAPI. To properly separate the signals emitted by the fluorescent dyes, sequential imaging was performed in two channels. The total number of dead spores (red ovals) was counted at a 514-nm excitation wavelength and detection bandwidth used for viewing PI staining. At least sixteen fields of view were analysed for each sample. All experiments were carried out at a concentration of 1.6 × 10^7^ spores/mL.

### Study of porphyrin bioactivity

The spores obtained from the honeybee colonies with the highest number of live *N. ceranae* spores were used for the porphyrin activity investigations. Ten porphyrins, TTMePP, TPyP, PP[Asp(ONa)_2_]_2_, PP[Glu(ONa)_2_]_2_, PP[Lys-ONa]_2_, PP[Lys-TFA]_2_, PP[Asp-Glu]_2_, PP[Lys-Lys]_2_, PP[Lys-Asp]_2_, and ZnPP[Asp(ONa)_2_]_2_, were analysed at initial concentrations of 10, 50 and 100 µM. The stock solutions of the porphyrins in water (2 mM) were sterilized by filtration through a sterile filter (pore size 0.22 μm) and stored at 4 °C in complete darkness until they were added to spores resuspended in 0.5% sucrose solution (3 × 10^7^ spores/mL). The experiments were performed in triplicate. Each replicate contained a set of 11 samples, including a reference control (without porphyrin). The samples, which consisted of 200 µL of the spore suspension and 800 µL of 0.5% sucrose solution (with or without a porphyrin), were incubated at 30 °C for 24 h in the dark or under visible light in 4-mL glass screw-top vials equipped with magnetic stirring bars. In a standard photoactivation procedure, the transparent glass vials containing microsporidia were evenly irradiated by four visible fluorescent lamps (Osram Lumilux, Cool White) with a controlled intensity of light (irradiance of 140 μmol/m^2^ s) and temperature (30 °C).

After 8 and 24 h of incubation, the number of spores was estimated using a light microscope according to the method described in Sect. 'Selection of honeybee colonies with the highest number of live *Nosema* spores'.

Additionally, PI-stained preparations of non-destroyed spores were made for each sample to determine survival of the spore after treatment with the porphyrins. We stained with only PI because the excitation wavelength of 405 nm used for viewing the DAPI stain is also in the range of the Soret band in the UV–Vis spectra of porphyrins. The staining procedure was carried out using the method described in Sect. 'Isolation of microsporidia and the determination of spore viability'. Survival is expressed as a percentage relative to that of a control sample containing no porphyrin taken at the beginning of each experiment (prior to the incubation) and after 24 h of incubation.

### Study of porphyrin uptake by microsporidia

Suspensions of *Nosema* spores with two different content of live spores were prepared and incubated in the dark with PP[Lys-TFA]_2_ and PP[Lys-Lys]_2_ to determine the differences in the uptake of the selected porphyrins. For this purpose, a suspension containing dead spores (2.8% of live spores) was prepared by heat inactivation of the spores at 80 °C for 1.5 h^27^. The dead spores were centrifuged (2000 rpm, 5 min), suspended in 1.5 mL of 0.5% sucrose solution containing the porphyrins (at a concentration of 50 µM) and incubated at 30 °C for 2 and 4 h. In parallel, freshly harvested spores with high content of live spores [90.4%; colony no C5(78)] were incubated with the selected porphyrins under the same conditions. Next, the spore suspension was centrifuged for 15 min at 4000 rpm and thoroughly washed using sterile aqueous 0.9% sodium chloride solution and then PBS buffer to remove any porphyrin residues. The procedure was repeated four times to remove porphyrin molecules that could be attached to the spore walls. The pelleted spores were diluted to give a final concentration of 10^6^ spores per mL and imaged using a confocal microscope under a 63× objective.

The auto-fluorescence of *Nosema* spores was accounted for using spore suspensions that were not treated with a porphyrin. A 405-nm diode laser was used for excitation of the porphyrins. The percentage of spores containing porphyrin was counted (ovals emitting red fluorescence) using a PMT detector in the 600–700 nm range. For each porphyrin, at least 16 fields of view were counted, and the spores containing porphyrin were differentiated in each field. In at least four different spore regions, emission spectra of spores emitting red fluorescence were collected using spectral imaging to confirm the presence of porphyrin. A 32-channel GaAsP spectral detector was used for the fluorescence spectral imaging. Spectral images and emission spectra from the spores were collected using a 405-nm laser set at 3% power and a GaAsP detector working in the 411–692 nm range. The pinhole diameter was set to 1 AU. A colour-coded image composed of 32 images, with each image acquired with a separate narrow bandwidth was obtained, and the overall image represents the complete spectral distribution of the fluorescence signals for every point in the microscopy image. The percent values were calculated against the mean number of microsporidia in DIC imaging mode.

### Effect of the selected porphyrins on the course of *Nosema ceranae* infection in honeybees

One-day post-emergence, *Nosema*-free honeybees (confirmed by PCR) were allocated into 40 wooden cages, each occupied by 40 bees, and maintained in laboratory conditions (1st day of the experiment). Three days post-emergence, honeybees in half of the cages were infected with *N. ceranae*, as described by Ptaszyńska et al.^23^. They were fed sucrose-water syrup containing *N. ceranae spores* (5 × 10^6^ spores/mL) isolated from colony C5*.* The infection procedure was performed over 2 days. On day 8 of the experiment, both infected and uninfected honeybees were divided into four experimental groups (one control and three porphyrin-treated groups) with 5 cages each. The porphyrin-treated groups were given sucrose-water syrup (1:1) supplemented with porphyrins: PP[Lys-TFA]_2_, PP[Lys-Lys]_2_, and ZnPP[Asp(ONa)_2_]_2_ (at concentrations of 100 μM). Control bees (in the infected and uninfected groups) were fed only with pure sucrose syrup, which was administered to the honeybees via syringes containing 5 mL of the syrup. To verify the level of infection, i.e., the number of *N. ceranae* spores, every second day (days 8, 10, 12, 14, 16, and 18 of the experiment), one honeybee from each of the five cages per experimental group was homogenized in 1 mL of sterile, distilled water and the number of *N. ceranae* spores was determined according to the method described by Hornitzky^55^ and Fries et al.^56^. To check whether the bees in the uninfected groups did not become accidentally infected with *N. ceranae*, one bee from each of the cages was analysed individually on days 8, 12, and 18. Additionally, throughout the whole experiment, dead bees were recorded daily and feed intake (determined as the amount of syrup consumed per individual bee per day) was measured every second day (Fig. S4) in specific groups.

### Statistics

Statistical analysis was performed using TIBCO Statistica version 13.3 (TIBCO Software Inc, US). Comparisons among the effect of the selected porphyrins on the *N. ceranae* spore number and viability were performed using one-way ANOVA, multiple comparisons with the control group were made using the Dunnett method for porphyrins at 10 and 50 µM. Before analysis, the normality of data distribution in each test group was examined using the Shapiro–Wilk test (*p* = 0.1 to *p* = 0.45). The uniformity of the variance was checked using the Leven test. For porphyrins at 100 M concentration, the results did not meet the assumptions of the parametric test, and heteroscedasticity appeared. Therefore, the results were analyzed using the ANOVA Kruskal–Wallis test, and significance between individual groups was obtained using comparisons of the mean ranks for all samples.

The analysis of the effect of the porphyrin compounds on the course of *N. ceranae* infection in honeybees on particular days were analysed by two-way ANOVA and Tukey’s post hoc test. Normality of data distribution was tested using the Shapiro–Wilk test (*p* = 0.06 to *p* = 0.08) and a categorized normality graph. The uniformity of the variance was checked using the Levene test. The mortality of honeybees was analysed by creation of Kaplan–Meier survival curves for the bees in each treatment. The curves were compared using a log-rank/Mantel-Cox post hoc test to determine which curves were significantly different from one another.

## Conclusions

These studies highlight the effect of amino acid identity on porphyrin activity and show that PPIX modification with aspartate and lysine are especially beneficial, whereas glutamate moieties do not improve the activity of porphyrins against *Nosema ceranae*. The presence of the metal ion in the porphyrin core and the type of counterion for the amino acid functional groups can also dramatically influence their activity on *Nosema* cells and toxicity to honeybees. It was confirmed that non-metallated porphyrins do not require light irradiation in their mechanism of action against microsporidia. Importantly, confocal microscopy analysis showed that the induced damage proceeds from the active uptake of the porphyrin by live spores and that the porphyrin is evenly distributed throughout the cells. Additionally, cell uptake is influenced by the length of the amino acid moiety used as a hydrophilic head group. Among the compounds evaluated, the porphyrins that most efficiently destroyed the microsporidia appeared to be PPIX derivatives bearing peptides with two (or more) amino acids, such as PP[Lys-Asp]_2_ and PP[Lys-Lys]_2_, which facilitated cellular uptake of the porphyrin. Thus, a balance between the hydrophobicity and hydrophilicity of amphiphilic porphyrins is an important consideration when designing effective microsporidia treatments and anti-*Nosema*-specific agents based on porphyrin ring systems. Further modification of the porphyrin periphery and chain elongation via coupling with amino acids to allow penetration of the impermeable spore exo- and endosporium should improve the activity of PPIX derivatives. In particular, lysine linked to PPIX is an ideal candidate for the future development of porphyrin-based antimicrosporidial agents because this amino acid can easily be functionalized via its amino or carboxylic acid moieties.

## Supplementary information

Supplementary Information.
