# Supplementary material for: Bioactivity studies of porphyrinoids against microsporidia isolated from honeybees
Source: Sci Rep. 2020 Jul 14;10:11553. doi: 10.1038/s41598-020-68420-5 (PMC7360595; doi:10.1038/s41598-020-68420-5)
Supplement: Supplementary file 1 — Supplementary Information. [file 41598_2020_68420_MOESM1_ESM.docx]

**Bioactivity studies of porphyrinoids against microsporidia isolated from honeybees**

Katarzyna Buczek^1^, Mariusz Trytek^1^*, Kamil Deryło^2^, Grzegorz Borsuk^3^, Katarzyna Rybicka-Jasińska^4^, Dorota Gryko^4^*, Małgorzata Cytryńska^5^, Marek Tchórzewski^2^

^1^Department of Industrial and Environmental Microbiology, Institute of Biological Sciences, Faculty of Biology and Biotechnology, Maria Curie-Skłodowska University, Akademicka 19, 20-033 Lublin, Poland; e-mail: katarzyna.buczek@poczta.umcs.lublin.pl (Katarzyna Buczek)

^2^Department of Molecular Biology, Institute of Biological Sciences, Faculty of Biology and Biotechnology, Maria Curie-Skłodowska University, Akademicka 19, 20-033 Lublin, Poland; e-mail: kamil@hektor.umcs.lublin.pl (Kamil Deryło), e-mail: maro@hektor.umcs.lublin.pl (Marek Tchórzewski)

^3^Institute of Biological Basis of Animal Production; Faculty of Biology, Animal Sciences and Bioeconomy; University of Life Sciences in Lublin, Akademicka 13, 20-950 Lublin, Poland; e-mail: grzegorz.borsuk@up.lublin.pl

^4^Institute of Organic Chemistry, Polish Academy of Sciences, Kasprzaka 44/52, 01-224 Warsaw, Poland;e-mail: dorota.gryko@icho.edu.pl (Dorota Gryko), e-mail:katarzyna.rybickajasinska@gmail.com (Katarzyna Rybicka-Jasińska)

^5^Department of Immunobiology, Institute of Biological Sciences, Faculty of Biology and Biotechnology, Maria Curie-Skłodowska University, Akademicka 19, 20-033 Lublin, Poland; e-mail: cytryna@poczta.umcs.lublin.pl (MałgorzataCytryńska)

*Corresponding author

E-mail 1: [mariusz.trytek@umcs.pl](mailto:mariusz.trytek@umcs.pl); e-mail 2: [mtrytek1@o2.pl](mailto:mtrytek1@o2.pl)(MT)

Department of Industrial and Environmental Microbiology, Institute of Biological Sciences, Maria Curie-Skłodowska University, 20-033 Lublin, Akademicka 19

Fax: +48-815375959; tel. +48-81-537-5958

**Supplementary material S1**

**SYNTHESIS**

**Zn-PP[Asp(OMe)-OMe]_2_**

To a 25-mL round bottomed flask equipped with a reflux condenser was added a solution of 30 mg of **PP[Asp(ONa)-ONa]_2_**(0.035 mmol) in 25 mL of chloroform. The reaction mixture was brought to reflux, and excess (5 mL) saturated methanolic zinc acetate solution was added. After 2 h, the mixture was cooled to room temperature, and the solvent was removed in vacuo to give a purple solid, which was dissolved in 50 mL of CH_2_Cl_2_ and washed with brine (3x50 mL). The organic phase was dried (Na_2_SO_4_), and the solvent was removed under reduced pressure. The obtained crude product (**Zn-PP[Asp(OMe)-OMe]_2_**) was purified by column chromatography (eluent: CH_2_Cl_2_:MeOH 98:2 (v/v/) mixture) to give 30 mg (0.033 mmol, 95% yield) of **Zn-PP[Asp(OMe)-OMe]_2_** as purple crystals.

^1^H NMR (500 MHz, CDCl_3_): δ = 9.30 (s, 1H, *meso*), 9.19 (s, 1H, *meso*), 8.84 (s, 1H, *meso*), 8.70 (s, 1H, *meso*), 8.14-8.04 [m, 2H, -CH=(vinyl)], 6.69-6.64 (m, 2H, -NH-), 6.29 [dd, *J* = 17.8, 9.5 Hz, 2H, =CH_2_ (vinyl)], 6.16 [dd, *J* = 11.5, 9.5 Hz, 2H, =CH_2_ (vinyl)], 4.31-4.26 (m, 2H, -CH-), 4.19-4.06 (m, 2H, por-CH_2_-), 4.06-3.92 (m, 2H, por-CH_2_-), 3.52 (s, 3H, por-CH_3_), 3.43 (s, 3H, por-CH_3_), 3.35 (s, 3H, por-CH_3_), 3.29-3.27 (m, 6H, -COOCH_3_, -CH_2_-CONH-), 3.24 (s, 3H, por-CH_3_), 2.92-2.90 (m, 2H, -CH_2_-), 2.67-2.59 (m, 2H, -CH_2_-), 1.79-1.50 (m, 8H, -COCH_3_, -CH_2_-), 1.30-1.32 (m, 1H, -CH-), 0.48-0.36 (m, 1H, -CH-) ppm.

^13^C NMR (125 MHz, CDCl_3_): δ = 172.5, 172.5, 170.7, 170.7, 169.9, 169.8, 146.8, 146.3, 146.2, 146.1, 145.7, 145.7, 145.5, 145.0, 138.2, 137.9, 136.2, 136.2, 136.1, 135.8, 135.8, 135.4, 130.6, 130.6, 119.3, 119.2, 97.3, 96.8, 96.4, 95.9, 52.1, 52.1, 50.1, 50.0, 47.5, 47.4, 39.1, 34.5, 34.4, 34.4, 29.7, 22.8, 22.8, 12.7, 12.6, 11.4, 11.2 ppm.

HRMS-ESI: m/z [M+Na]^+^ calcd for C_46_H_50_O_10_N_6_Zn: 933.2778; found: 933.2764.

UV/Vis (MeOH): λ_max_ (ε) = 415 (0.67 x 10^5^), 544 (1.08 x 10^4^), 586 nm (1.05 x 10^3^).

**Zn-PP[Asp(ONa)-ONa]_2_**

**Zn-PP[Asp(OMe)-OMe]_2_**(30 mg, 0.033 mmol) was dissolved in CH_2_Cl_2_ (4 mL), and the resulting solution was heated to 40 °C. Then, MeOH (4 mL) and 4 M aq. NaOH (2 mL) were sequentially added, and the mixture was refluxed until the reaction was complete (TLC, 3 h). The volatile organic compounds were removed under reduced pressure, and the suspension was placed in a vial for subsequent centrifugation. Crystallization (H_2_O-MeOH) followed by washing several times with MeOH and then Et_2_O gave a red solid; yield 27 mg (95%).

^1^H NMR (500 MHz, TFA-d_1_): δ = 11.14-11.07 (m, 4H, *meso*), 8.36-8.28 (m, 2H), 6.65 [dd, *J* = 10.5, 5.1 Hz, 2H, =CH_2_ (vinyl)], 6.46 [dd, *J* = 18.3, 6.5 Hz, 2H, =CH_2_ (vinyl)], 4.87-4.67 (m, 6H, -CH-), 4.32-4.23 (m, 2H, -CH-), 3.93-3.76 (m, 13H, -CH_2_-CO-NH-, por-CH_3_), 3.41-3.00 (m, 8H, -CH-, por-CH_3_), 1.38-1.34 (m, 3H, -CH_2_-) ppm.

^13^C NMR (125 MHz, TFA-d_1_): δ = 176.2, 174.6, 170.9, 170.8, 162.8, 162.4, 162.3, 162.3, 162.1, 161.9, 161.9, 161.6, 161.6, 161.3, 161.3, 161.2, 160.9, 142.5, 142.1, 141.9, 141.9, 141.5, 140.9, 140.7, 139.9, 139.7, 128.1, 126.7, 126.2, 118.0, 117.6, 117.2, 115.7, 115.3, 114.9, 113.5, 113.1, 112.7, 111.2, 110.8, 110.4, 110.0, 99.8, 65.9, 49.7, 35.9, 33.3, 21.5, 12.4, 10.8, 10.5 ppm.

HRMS-ESI: m/z [M+Na]^+^ calcd for C_42_H_42_O_10_N_6_Zn: 877.2152; found: 877.2144.

UV/Vis (H_2_O): λ_max_ (ε) = 413 (0.12 x 10^5^), 547 (1.64 x 10^4^), 582 nm (1.75 x 10^3^).

**PP[Lys(TFA)-Asp(OH)-OH]_2_**was synthesized manually by Fmoc chemistry on a 0.02-mmol scale. Fmoc-Lys(Boc) (4 equiv.) was attached to Wang resin (Fmoc-Asp-Wang resin), and PP(IX) was subsequently attached to the Fmoc-Lys(Boc)-Glu-Wang resign. Fmoc deprotection was performed with 20% piperidine in DMF (1.5 mL, 1-2 h), and coupling was achieved with HBTU (6 equiv.) and DIPEA (6 equiv.) in DMF (2 mL). After the final coupling, the resin was washed with DMF (5 x 1 mL) and DCM (5 x 1 mL) and dried. The product was cleaved from the resin with TFA/DCM (25%, v/v) with a catalytic amount of anisole in 3 h. The obtained crude product (**PP[Lys(TFA)-AspOH)-OH]_2_**) was precipitated with Et_2_O, centrifuged and subsequently purified by RP column chromatography (eluent: H_2_O:MeOH:TFA 95:94.5:0.5 (v/v/v) mixture).

^1^H NMR (500 MHz, TFA-d_1_): δ = 10.32-10.22 (m, 4H, *meso*), 8.55-8.37 [m, 4H, -CH=(vinyl)], 8.06-8.02 (m, 2H), 7.72 (br s, 4H, NH), 6.46 (d, *J* = 17.8 Hz, 2H, -CH-), 6.24 (d, *J* = 11.4 Hz, 2H, -CH-), 4.75-4.65 (m, 2H, -CH-), 4.37-4.27 (m, 4H, -CH-), 4.11-4.02 (m, 2H, -CH_2_-), 3.73 (d, *J* = 9.9 Hz, 6H, por-CH_3_), 3.63 (d, J = 11.1 Hz, 6H, por-CH_3_), 3.51-3.21 (m, 6H, -COOCH_3_, -CH_2_-CONH-), 3.14-3.07 (m, 4H, -CH-, -CH_2_), 2.71-2.64 (m, 4H, -CH, -CH_2_), 2.39-2.30 (m, 2H), 1.68-1.61 (m, 2H, -CH-), 1.57-1.40 (m, 7H, -CH-, -CH_2_), 1.31-1.25 (m, 3H, -CH-), -3.8 (br s, 2H) ppm.

^13^C NMR (125 MHz, DMSO-d_6_): δ =173.4, 171.9, 171.7, 170.6, 157.9, 130.0, 121.2, 97.5, 97.1, 51.9, 49.6, 48.5, 40.1, 40.0, 39.3, 39.2, 39.1, 39.0, 38.5, 38.2, 36.3, 30.4, 26.4, 22.0, 21.8, 12.6, 12.6, 11.3 ppm.

HRMS-ESI: m/z [M+H]^+^ calcd for C_54_H_69_O_12_N_10_: 1049.5096; found: 1049.5098.

UV/Vis (MeOH): λ_max_ (ε) = 401 (7.80 x 10^4^), 506 (7.84 x 10^3^), 540 (8.41x 10^3^), 576 (5.22 x 10^3^), 627 (3.63 x 10^3^).

**PP[Lys(TFA)-Lys(TFA)-OH]_2_**was synthesized manually by Fmoc chemistry on a 0.02-mmol scale. Fmoc-Lys(Boc) (4 equiv.) was attached to Wang resin (Fmoc-Lys-Wang resin), and PP(IX) was subsequently attached to the Fmoc-Lys(Boc)-Glu-Wang resign. Fmoc deprotection was performed with 20% piperidine in DMF (1.5 mL, 1-2 h), and coupling was achieved using HBTU (6 equiv.) and DIPEA (6 equiv.) in DMF (2 mL). After the final coupling, the resin was washed with DMF (5 x 1 mL) and DCM (5 x 1 mL) and dried. The product was cleaved from the resin with TFA/DCM (25%, v/v) with a catalytic amount of anisole in 3 h. The obtained crude product (**PP[Lys(TFA)-Lys(TFA)-OH]_2_**) was precipitated with Et_2_O, centrifuged and subsequently purified by RP column chromatography (eluent: H_2_O:MeOH:TFA 95:94.5:0.5 (v/v/v) mixture).

**^1^H NMR** (500 MHz, TFA-d_1_): δ = 10.41-10.24 (m, 4H, *meso*), 8.59-8.49 [m, 2H, -CH=(vinyl)], 8.17-8.10 [m, 4H, -CH=(vinyl), =CH-], 7.82-7.55 (m, 16H, -CH-, -CH_2_), 6.48 (dd, J = 19.7, 6.7 Hz, 2H, -CH_2_), 6.25 (d, J = 11.5 Hz, 2H, -CH_2_), 4.39-4.26 (m, 5H, -CH-, -CH_2_), 4.12-4.05 (m, 2H, -CH-), 3.76 (d, *J* = 9.25 Hz, 6H, por-CH_3_), 3.64 (d, J = 12.9 Hz, 6H, por-CH_3_), 3.56-3.42 (m, 2H, -CH-), 3.21-3.10 (m, 4H, -CH-, -CH_2_), 2.81-2.69 (m, 6H, -CH-, -CH_2_), 2.40-2.31 (m, 3H, -CH-), 1.69-1.61 (m, 2H, -CH-), 1.59-1.41 (m, 5H, -CH-, -CH_2_), 1.39-1.28 (m, 5H, -CH-, CH_2_), 1.23-1.14 (m, 2H, -CH-), 1.06-0.96 (m, 2H, -CH-), -3.78 (br s, 2H, NH) ppm.

**^13^C NMR** (125 MHz, DMSO-d_6_): δ =173.3, 171.7, 158.1, 158.1, 157.9, 157.8, 130.0, 130.0, 121.3, 118.3, 115.9, 115.9, 113.4, 112.6, 54.5, 52.1, 51.6, 40.1, 40.0, 39.9, 39.8, 39.8, 39.7, 38.5, 38.3, 38.1, 31.4, 30.2, 30.2, 26.5, 26.4, 22.3, 21.9, 21.8, 12.6, 12.6, 11.4 ppm.

**HRMS-ESI**: m/z [M+H]^+^ calcd for C_58_H_83_O_8_N_12_: 1075.6457; found: 1075.6451.

**UV/Vis** (MeOH): λ_max_ (ε) = 401 (9.40 x 10^4^), 504 (7.94 x 10^3^), 535 (6.44 x 10^3^), 576 (4.11 x 10^3^), 627 (2.73 x 10^3^).

**^1^H NMR and ^13^C NMR SPECTRA of THE PORPHYRINS**

**Zn-PP[Asp(OMe)-OMe]_2_**

**Zn-PP(Asp(ONa)-ONa)_2_**

**__**

**__**

**PP[Lys(TFA)-Asp(OH)-OH]_2_**

**PP[Lys(TFA)-Lys(TFA)]_2_**

**Supplementary material S2 Table.**

Number of *Nosema* spp. spores in different honeybee colonies assayed from winter beehive debris. I, II and III represent replicates of the assays.

| **Colony** | **Number of *Nosema* sp. spores per bee [×10^6^]** | | | **Mean** | **Standard deviations** |
| --- | --- | --- | --- | --- | --- |
|  | **I** | **II** | **III** |  |  |
| **CCA** | 49 | 47.6 | 47.4 | 48 | 0.87 |
| **3** | 0 | 0 | 0 | 0 | 0 |
| **8** | 17.6 | 18.2 | 17 | 17.6 | 0.6 |
| **36** | 26.7 | 23.8 | 26.3 | 25.6 | 1.57 |
| **50** | 14.3 | 14.5 | 14.4 | 14.4 | 0.1 |
| **51** | 17.7 | 17 | 18.1 | 17.6 | 0.56 |
| **52** | 120.7 | 100.3 | 119.8 | 113.6 | 11.53 |
| **53** | 23.5 | 25.5 | 23 | 24 | 1.32 |
| **54** | 222.9 | 222.1 | 221 | 222 | 0.44 |
| **55** | 20.3 | 18.3 | 19 | 19.2 | 1.01 |
| **59** | 3.2 | 4 | 3.9 | 3.7 | 0.95 |
| **60** | 157.6 | 158.9 | 158.7 | 158.4 | 0.66 |
| **61** | 0 | 0 | 0 | 0 | 0 |
| **65** | 122.6 | 125.7 | 129.8 | 126 | 3.62 |
| **66** | 0 | 0 | 0 | 0 | 0 |
| **67** | 11.3 | 9.2 | 9.5 | 10 | 1.14 |
| **69** | 126 | 125.9 | 127.3 | 126.4 | 0.78 |
| **70** | 251.3 | 256.9 | 241.8 | 250 | 7.63 |
| **71** | 0 | 0 | 0 | 0 | 0 |
| **76** | 0 | 0 | 0 | 0 | 0 |
| **77** | 628.9 | 650 | 631.5 | 636.8 | 11.51 |
| **78** | 289.2 | 288.5 | 286.3 | 288 | 1.51 |
| **79** | 0 | 0 | 0 | 0 | 0 |
| **82** | 32.8 | 31.6 | 31.6 | 32 | 0.69 |
| **84** | 25 | 27.8 | 24 | 25.6 | 1.97 |
| **90** | 15.4 | 10 | 13 | 12.8 | 2.71 |

**Supplementary material S3 Figure**

Experimental procedures.


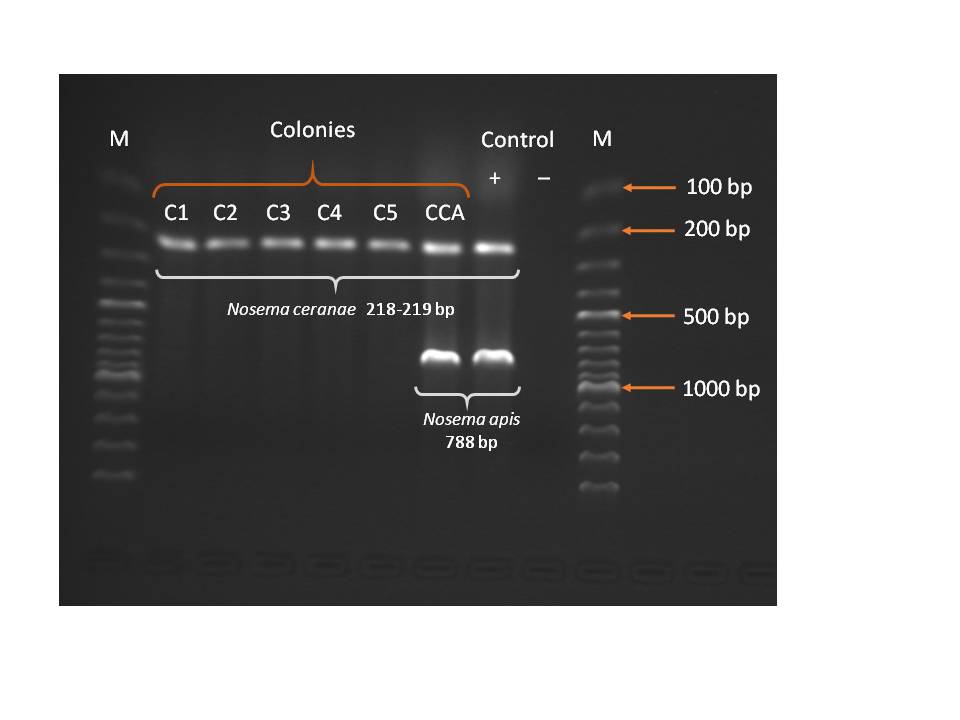
2% agarose gels showing PCR products amplified from *Nosema ceranae* DNA extracted from spores isolated from winter beehive debris of selected honeybee colonies (C1-C5).

M: Molecular weight markers (Gene ruler, 1-kb ladder, Thermo Scientific).

Lanes C1-C5 correspond to the colonies with the highest *Nosema* infection rates. CCA represents a honeybee colony with a moderate infection rate.

**Supplementary material S4 Figure**

**
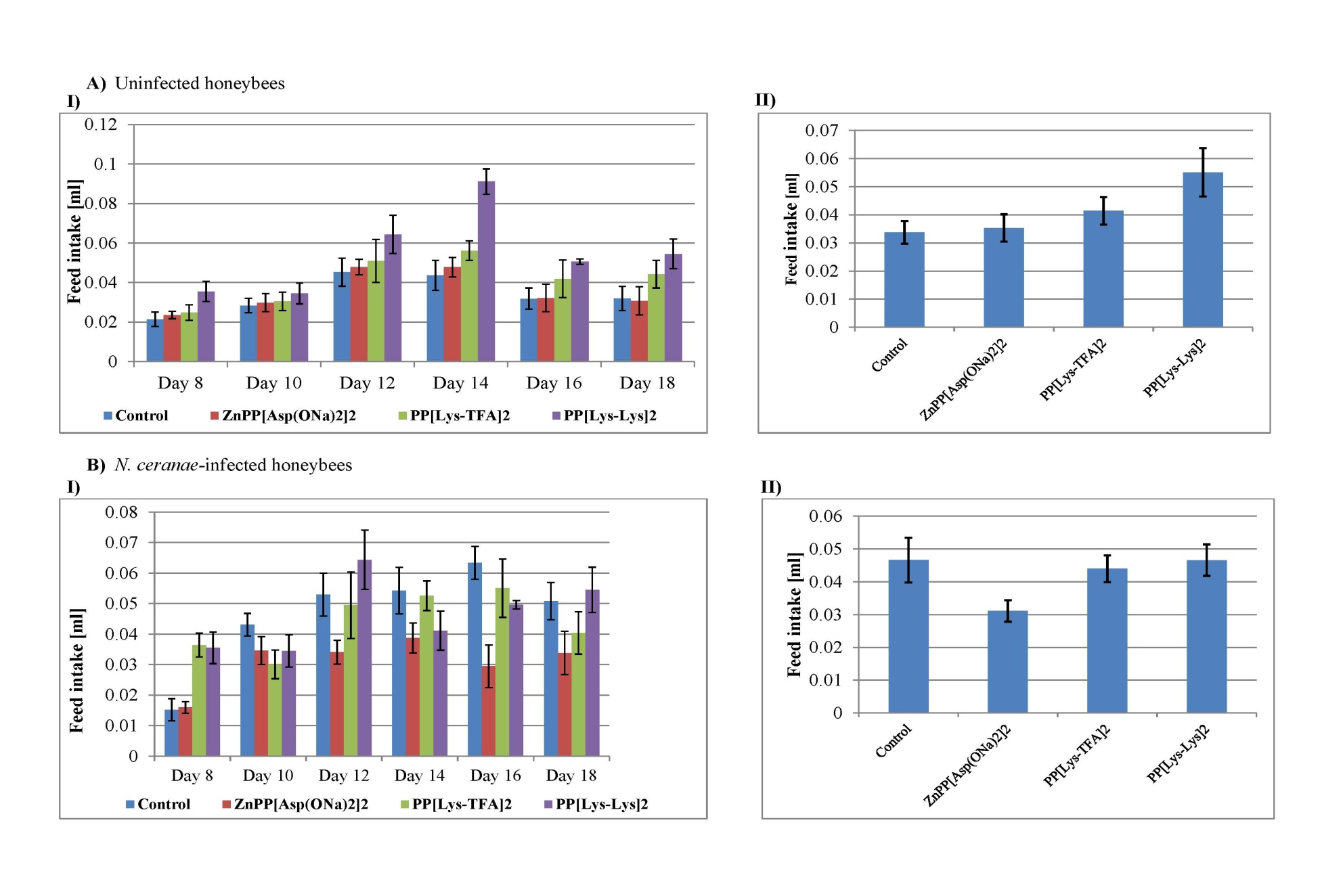
**
